# Self‐Organizing Ovarian Somatic Organoids Preserve Cellular Heterogeneity and Reveal Cellular Contributions to Ovarian Aging

**DOI:** 10.1111/acel.70333

**Published:** 2025-12-29

**Authors:** Shweta S. Dipali, Aubrey Converse, Madison Q. Gowett, Pratik Kamat, Emily J. Zaniker, Abigail Fennell, Teresa Chou, Michele T. Pritchard, Mary Zelinski, Jude M. Phillip, Francesca E. Duncan

**Affiliations:** ^1^ Department of Obstetrics and Gynecology, Feinberg School of Medicine Northwestern University Chicago Illinois USA; ^2^ Department of Chemical and Biomolecular Engineering, Institute for Nanobiotechnology Johns Hopkins University Baltimore Maryland USA; ^3^ Department of Biomedical Engineering Johns Hopkins University Baltimore Maryland USA; ^4^ Department of Pharmacology, Toxicology, and Therapeutics University of Kansas Medical Center Kansas City Kansas USA; ^5^ Institute for Reproductive and Developmental Sciences University of Kansas Medical Center Kansas City Kansas USA; ^6^ Division of Reproductive & Developmental Sciences Oregon National Primate Research Center Beaverton Oregon USA; ^7^ Department of Oncology, Sidney Kimmel Comprehensive Cancer Center Johns Hopkins School of Medicine Baltimore Maryland USA

**Keywords:** actin, cellular stiffness, organoid, ovary, reproductive aging, stroma

## Abstract

Ovarian somatic cells are essential for reproductive function, but no existing ex vivo models recapitulate the cellular heterogeneity or interactions within this compartment. We engineered an ovarian somatic organoid model by culturing a stroma‐enriched fraction of mouse ovaries in scaffold‐free agarose micromolds. Self‐organized ovarian somatic organoids maintained diverse cell populations, produced extracellular matrix, and secreted hormones. Organoids generated from reproductively old mice exhibited reduced aggregation and growth compared to young counterparts, as well as differences in cellular composition. Interestingly, matrix fibroblasts from old mice demonstrated upregulation of pathways associated with the actin cytoskeleton and downregulation of cell adhesion pathways, indicative of increased cellular stiffness that may impair organoid aggregation. Cellular morphology, which is regulated by the cytoskeleton, significantly changed with age and in response to actin modulation. Moreover, actin modulation altered organoid aggregation efficiency. Overall, ovarian somatic organoids have advanced knowledge of cellular contributions to ovarian aging.

## Graphical abstract

We report the development and application of a mouse ovarian somatic organoid model to elucidate a fundamental new understanding of the cell biology of the aging ovarian microenvironment.

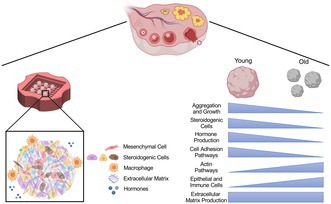

## 1 Introduction

Ovarian follicles, comprised of oocytes and their surrounding somatic support cells, are the functional units of the ovary and are essential for producing gonadal hormones as well as fertilization‐competent gametes. Ovarian follicles reside and develop in a complex and heterogenous stromal microenvironment composed of fibroblasts, immune cells, epithelial cells, interstitial fibroblasts and mesenchymal cells, blood and lymphatic vessels, nerves, and a dynamic extracellular matrix (ECM) (Kinnear et al. 2020). Broadly defined stromal cells, encompassing all of the subpopulations present in this microenvironment, are critical for supporting follicle development as co‐culture of primary and early secondary follicles with feeder layers of macrophage and theca‐enriched stromal cells improves follicle development (Tingen et al. 2011).

The structure and function of the ovarian stroma are significantly altered during reproductive aging (Furuya 2012; Kinnear et al. 2020; Shen et al. 2023). Ovaries from reproductively old mice have a greater proportion of immune and epithelial cells, as well as fewer broadly classified fibroblastic stromal cells than young counterparts (Isola et al. 2024). Additionally, the ovarian stroma becomes inflammatory with advanced age and is associated with altered macrophage ontogeny and polarization, as well as the presence of a unique population of multinucleated macrophage giant cells (Briley et al. 2016; Converse et al. 2025; Foley et al. 2021; Zhang et al. 2025, 2020). There is also an age‐dependent shift in ovarian immune cell populations with a skew towards adaptive immunity (Ben Yaakov et al. 2023; Isola et al. 2024). The relative abundance of subpopulations of ovarian fibroblasts changes with age, as does their transcriptome (Isola et al. 2024; Landry et al. 2022). Reproductive aging results in greater proportions of senescence‐associated secretory phenotype (SASP) fibroblasts in the ovarian stroma, as well as decreased expression of genes involved in collagen degradation, which may contribute to age‐related ovarian fibrosis and tissue stiffness (Amargant et al. 2020; Briley et al. 2016; Grzeczka et al. 2025; Isola et al. 2024; Landry et al. 2022; Umehara et al. 2022). In addition to collagens, the composition of the broader ovarian ECM is altered with age (Dipali et al. 2023; Ouni et al. 2022).

Given the importance of the stroma in ovarian physiology and aging, robust in vitro strategies are needed to study this compartment in a controlled manner. However, cultures of specific ovarian cell populations inherently do not capture the cellular heterogeneity and diverse cell–cell and cell‐matrix interactions of the ovarian stroma in vivo (Gamwell et al. 2012). Although primary somatic cells isolated from a stroma‐enriched fraction of the mouse ovary are initially heterogenous, two‐dimensional culture results in expansion of the macrophage population (Tingen et al. 2011). Ovarian tissue explants maintain the cellular heterogeneity and structural organization of the native tissue, but they can vary in composition due to where within the tissue the explants were derived (Mara et al. 2020; Zink et al. 2018). Organoids, defined as three‐dimensional, multi‐cellular, miniaturized versions of organs or tissues, have been utilized to model several reproductive tissues and address the shortcomings of traditional cell culture and tissue explants (Alzamil et al. 2021; Haider and Beristain 2023). Most current ovarian organoid models utilize a single cell type, model follicle structures rather than the ovarian stroma, or are focused on disease states such as ovarian cancer (Del Valle and de Chuva Sousa Lopes 2023; Hu et al. 2024; Kopper et al. 2019; Kwong et al. 2009; Pierson Smela et al. 2023). In tandem with this study, methods to culture human ovarian somatic cells in 3D culture have been developed, but have not been applied to mouse models or used in the context of aging (Di Nisio et al. 2025).

Therefore, the goals of this study were to develop an organoid model of the somatic compartment of the mouse ovary, primarily enriched for extrafollicular cell types, to inform mechanisms of ovarian aging. Using scaffold‐free agarose micromolds, we demonstrated that a stroma‐enriched fraction of ovarian somatic cells can self‐assemble into solid structures. Organoids contained all major somatic cell types of the ovary throughout culture, produced an ECM, and secreted hormones. However, ovarian somatic organoids generated using cells from reproductively old mice exhibited compromised aggregation, growth, and hormone production relative to young counterparts. The age‐dependent differences in aggregation capacity were likely due in part to changes at the cellular level because, compared to young mice, mesenchymal cells from reproductively old mice showed transcriptomic changes that are suggestive of an age‐dependent increase in cellular stiffness. To further investigate the age‐associated changes to cytoskeletal dynamics, we evaluated cell morphology and organoid aggregation in response to pharmacological actin modulation. We found that primary ovarian somatic cells exhibit heterogeneous morphology that changes with age and in response to actin depolymerization or stabilization. Actin depolymerization restored the aggregation potential of ovarian somatic cells from old mice, whereas actin stabilization reduced the aggregation of ovarian somatic cells from young mice, demonstrating that the actin cytoskeleton plays a role in organoid formation. Thus, this robust organoid model may be used to study the ovarian somatic compartment and has revealed cellular mechanisms underlying aging in the ovarian stroma.

## 2 Results

### 2.1 Ovarian Somatic Organoids Self‐Assemble and Contain Key Ovarian Cell Populations With Distinct Spatial Organization

**FIGURE 1:**
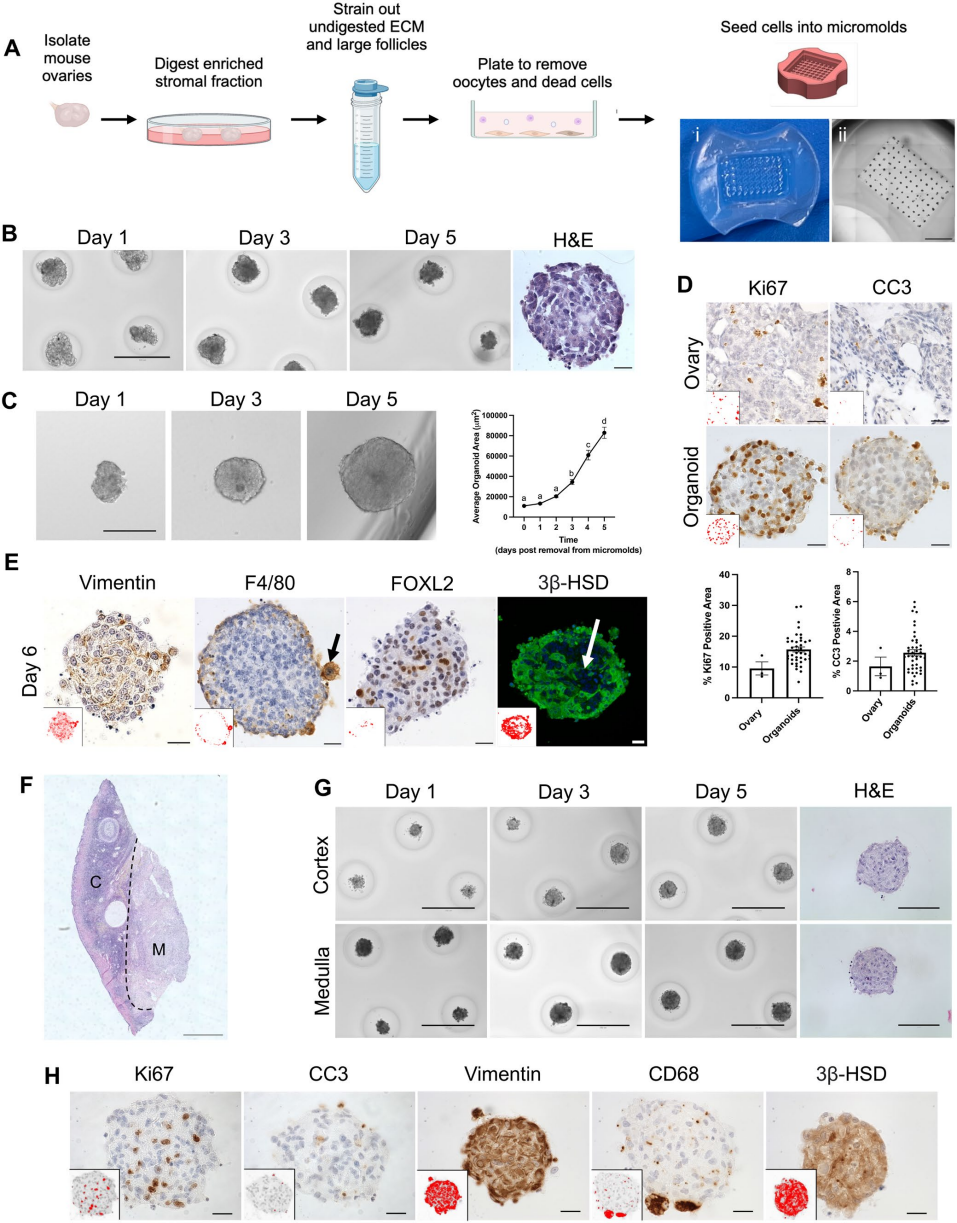
Ovarian somatic organoids self‐assemble into solid structures containing key ovarian cell populations with distinct spatial organization. (A) Schematic of workflow to isolate primary mouse ovarian somatic cells and generate organoids and images of an empty agarose micromold (i) and a scan of an agarose micromold following organoid generation (ii). (B) Representative transmitted light images of murine ovarian somatic organoids cultured in IntestiCult at Days 1, 3, and 5 of culture. *N* = 4 micromolds. Representative image of H&E‐stained murine organoid section following 5 days of culture in IntestiCult. Scale bars for transmitted light images = 400 μm and scale bars for H&E image = 20 μm. (C) Representative images of murine ovarian somatic organoids at Days 1, 3, and 5 of additional culture following removal from micromolds after 3 initial days of culture. Scale bars = 200 μm. Quantification of average organoid area over 5 additional days of culture following removal from micromolds. Error bars represent the standard error of the mean (SEM). *p* < 0.05 for a versus b and *p* < 0.0001 for all other comparisons by one‐way ANOVA. *N* = 28 organoids. (D) Representative images of Ki67 (brown) and cleaved caspase‐3 (CC3, brown) IHC staining of mouse ovarian tissue sections and murine ovarian somatic organoid sections. Nuclei were detected by hematoxylin staining (blue‐purple). Scale bars = 20 μm. Insets are thresholded images highlighting positive pixels. Quantification of the percent Ki67 and CC3 positive area in regions of interest in mouse ovaries (*N* = 3 ovaries) and ovarian somatic organoids after 5 days of culture (*N* = 40–44). Error bars represent the standard error of the mean (SEM). (E) Representative images of IHC labelling of murine ovarian organoid sections after 6 days of culture using antibodies against Vimentin (brown), F4/80 (brown), FOXL2 (brown), and 3β‐HSD (green). For chromogenic staining nuclei were detected by hematoxylin (blue‐purple), and for fluorescent staining nuclei were detected by DAPI (blue). Arrows show F4/80‐positive macrophages enriched at the perimeter of organoids (black) and 3β‐HSD‐positive steroidogenic cells consistently depleted from the core of organoids (white). Insets are thresholded images highlighting positive pixels. Scale bars = 20 μm. *N* = 39–49 organoids. (F) Representative scan of H&E‐stained rhesus macaque ovarian tissue. Dashed line indicates separation of cortex (C) from medulla (M). Scale bar = 200 μm. (G) Representative images of ovarian somatic organoids generated from rhesus macaque ovarian cortex (top) or medulla (bottom) at Days 1, 3, and 5 of culture. Representative images of H&E‐stained rhesus macaque organoid sections following 5 days in culture. Scale bars for transmitted light images = 400 μm and scale bars for H&E images = 100 μm. *N* = 2 biological replicates for ovarian somatic cell isolation and ovarian somatic organoid generation from rhesus macaque ovarian tissue, 1–2 micromolds per biological replicate. (H) Representative images of IHC labelling of rhesus macaque medullary organoid sections after 6 days of culture using antibodies against Ki67 (brown), cleaved caspase‐3 (CC3, brown), Vimentin (brown), CD68 (brown), and 3β‐HSD (brown). Nuclei were detected by hematoxylin staining (blue‐purple). Insets are thresholded images highlighting positive pixels. Scale bars = 20 μm. *N* ≥ 30 organoids.

Mouse ovarian somatic organoids were generated by digesting an enriched ovarian stromal fraction into a single cell suspension followed by filtering and differential plating to isolate viable primary ovarian somatic cells from oocytes (Figure 1A). These cells were then seeded into agarose micromolds to generate organoids in an ECM scaffold‐free manner (Figure 1A). Organoid formation was dependent on the number of cells seeded into the agarose micromold, with a larger number of cells initially seeded resulting in better aggregation and larger organoids (Figure S1A–C). Moreover, IntestiCult mouse intestinal organoid growth medium best supported organoid aggregation and growth relative to other commercially available media, including HepatiCult, MesenCult, and RPMI‐1640, resulting in fewer aggregates per microwell and larger organoid area compared to the other media tested (Figure 1B, Figure S1D–F). Therefore, 250,000 cells per agarose micromold and IntestiCult media were used for organoid generation and culture in all further experiments. The resulting organoids were solid structures that remained intact and continued to grow even when removed from the agarose micromolds (Figure 1B,C). Furthermore, organoids exhibited similar percentages of proliferating and apoptotic cells as the mouse ovary in vivo (organoids Ki67‐positive area: 15.7% ± 0.8%, ovary Ki67‐positive area: 9.5% ± 2.1%, *p* > 0.05; organoids cleaved caspase 3 (CC3)‐positive area: 2.6% ± 0.2%, ovary CC3‐positive area: 1.6% ± 0.6%, *p* > 0.05) (Figure 1D). Interestingly, while Ki67‐positive cells were distributed throughout the organoids, CC3‐positive cells were primarily restricted to the perimeter of organoids (Figure 1D).

To determine whether key ovarian cell types were present in these organoids, we performed immunohistochemistry using antibodies against vimentin (fibroblasts and myofibroblasts), alpha‐smooth muscle actin (⍺‐SMA; myofibroblasts and smooth muscle cells), desmin (smooth muscle cells), F4/80 (murine macrophages), FOXL2 (granulosa and granulosa‐lutein cells), and 3‐beta‐hydroxysteroid dehydrogenase (3β‐HSD; theca, theca‐lutein, and steroidogenic stromal cells) (Figure 1E; Figure S2). By Day 6 of culture, vimentin‐positive fibroblasts and FOXL2‐positive steroidogenic cells were present throughout ovarian somatic organoids, whereas myofibroblasts and smooth muscle cells marked by ⍺‐SMA and desmin were not present in the organoids (Figure 1E; Figure S2A–C,E). F4/80‐positive macrophages were restricted to the organoid perimeter, similar to CC3‐positive apoptotic cells (Figure 1D,E; Figure S2D). Interestingly, the F4/80‐positive cells were hypertrophied by Day 6 of culture, consistent with phagocytic activity (Figure 1E; Figure S2D). Moreover, 3β‐HSD‐positive cells self‐organized such that they were consistently depleted from the organoid core (Figure 1E; Figure S2F–H).

Significantly fewer vimentin‐positive fibroblasts were present in organoids at both Days 1 (23.8% ± 1.3%, *p* = 0.04) and 6 (24.6% ± 2.1%, *p* = 0.004) of culture compared to the mouse ovary (48.7% ± 10.3%) (Figure S2A). Although not statistically significant, fewer ⍺‐SMA‐positive cells were present in organoids at Day 1 of culture (5.5% ± 0.8%) compared to the mouse ovary (14.2% ± 2.3%), and these cells were mostly absent in organoids by Day 6 of culture (0.6% ± 0.2%, *p* < 0.0001) in comparison to Day 1 (Figure S2B). Additionally, desmin‐positive cells were largely absent in organoids at Days 1 (0.2% ± 0.1%) and 6 (0.1% ± 0.0%) of culture (Figure S2C). However, by Day 6 of culture, F4/80‐positive macrophages, in addition to FOXL2‐ and 3β‐HSD‐positive steroidogenic cells, were present in organoids in similar quantities to the mouse ovarian somatic compartment in vivo (Figure S2D–F). Overall, ovarian somatic organoids are composed of diverse cell populations which undergo dynamic changes during culture.

Of note, these methods were also used to generate organoids using primary ovarian somatic cells isolated from both the cortex and medulla of rhesus macaque ovaries (Figure 1F,G). Similar to mouse, ovarian somatic cells isolated from NHP ovaries self‐assembled to form organoids within 24 h (Figure S3A). NHP organoids contained Ki67‐positive proliferative cells throughout the structures, which were more abundant than CC3‐positive apoptotic cells (Figure 1H, Figure S3B). Thus, this technique has translatability to non‐human primates, humans, and other large mammalian species. Organoids generated from rhesus macaque ovarian tissue also contained vimentin‐positive fibroblast lineage cells, CD68‐positive macrophages, as well as 3β‐HSD‐positive steroidogenic cells (Figure 1H, Figure S3B). Although the spatial organization of several cell types, including Ki67‐positive proliferative cells, vimentin‐positive fibroblasts, and CD68‐positive macrophages appears to be similar in NHP organoids relative to mouse counterparts, whether all features are conserved requires further investigation.

### 2.2 Organoids Exhibit Age‐Dependent Differences in Aggregation, Growth, and Function

**FIGURE 2:**
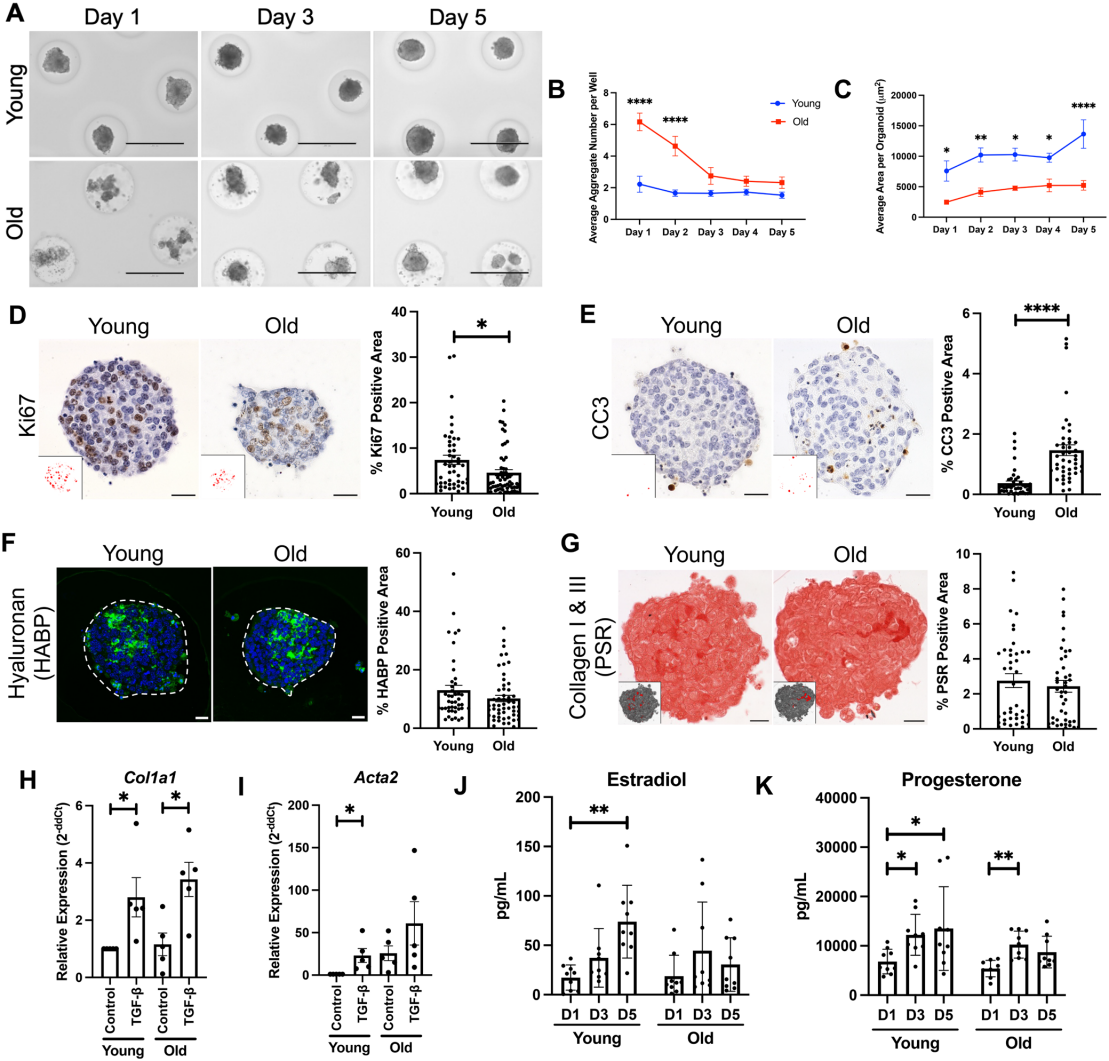
Organoids exhibit age‐dependent differences in aggregation, growth, and function. (A) Representative images of ovarian somatic organoids generated from reproductively young (6–12 weeks) or reproductively old (10–14 months) mice at days 1, 3, and 5 of culture. Scale bars = 400 μm. *N* = 4 micromolds per age. (B, C) Quantification of the average number of aggregates per microwell (B) and average area per organoid (C) over 5 days of culture for organoids generated from reproductively young and old mice. Error bars represent the standard error of the mean (SEM). *p < 0.05, **p < 0.01, and ****p < 0.0001 by two‐way ANOVA. *N* = 4 micromolds per age. (D, E) Representative images of Ki67 (D, brown) and cleaved caspase‐3 (CC3, E, brown) IHC staining of young and old ovarian somatic organoid sections. Nuclei were detected by hematoxylin staining (blue‐purple). Scale bars = 20 μm. Insets are thresholded images highlighting positive pixels. Quantification of the percent Ki67 (D) and CC3 (E) positive area in young (*N* = 42–46) and old (*N* = 44–57) ovarian somatic organoids after 5 days of culture. Error bars represent the standard error of the mean (SEM). *p < 0.05 and ****p < 0.0001 by Welch's *t*‐tests. (F) Representative images of hyaluronan binding protein (HABP, green) assays performed with young and old ovarian somatic organoid sections. Nuclei (blue) were detected with DAPI. Scale bars = 20 μm. Organoids are outlined by dashed lines. Quantification of the percent HABP‐positive area in young (*N* = 43) and old (*N* = 53) ovarian somatic organoids after 5 days of culture. Error bars represent the standard error of the mean (SEM). (G) Representative images of Picrosirius Red (PSR)‐stained young and old ovarian somatic organoid sections. Scale bars = 20 μm. Insets are thresholded images highlighting positive pixels. Quantification of the percent PSR‐positive area in young (*N* = 43) and old (*N* = 53) ovarian somatic organoids after 5 days of culture. Error bars represent the standard error of the mean (SEM). (H, I) Gene expression of *Col1a1* (H) and *Acta2* (I) in ovarian somatic organoids measured by RT‐qPCR following 48‐h treatment with 10 ng/mL TGF‐β or control. Gene expression for TGF‐β‐treated organoids was graphed as fold‐change over young control. *p < 0.05 by Welch's *t*‐tests after performing Shapiro–Wilk tests to confirm normality. *N* = 5 trials. (J, K) Estradiol (J) and progesterone (K) secretion measured in conditioned media of young and old ovarian somatic organoids by ELISAs at Days 1, 3, and 5 of culture. *p < 0.05 and **p < 0.01 by Kruskal–Wallis tests followed by Dunn's multiple comparisons tests to compare between time points for each age group separately. *N* = 9 micromolds over 3 trials.

Given that the ovarian stroma changes significantly with age in vivo, we wanted to determine whether age‐dependent differences were reflected in the ability of primary ovarian somatic cells to form organoids (Dipali et al. 2023; Isola et al. 2024). Organoids generated from reproductively old mice exhibited impaired aggregation as determined by quantifying the average number of aggregates per microwell, with a larger number indicative of poor aggregation potential (Figure 2A,B). There were more aggregates per microwell of the agarose molds in samples derived from reproductively old mice relative to young at both Day 1 and 2 of culture (old Day 1: 6.2 ± 0.6, young Day 1: 2.2 ± 0.5, *p* < 0.0001; old Day 2: 4.6 ± 0.6, young Day 2: 1.7 ± 0.2, *p* < 0.0001) (Figure 2A,B). Given that the same culture conditions were utilized to generate and culture organoids from both young and old mice, age‐dependent differences in organoid formation reflect alterations in cell‐intrinsic properties. Additionally, organoids generated from old mice were 46.6%–61.7% smaller in size across 6 days of culture compared to organoids from young mice (Figure 2A,C). Consistent with this smaller size, organoids generated from old mice had fewer Ki67‐positive proliferative cells and more CC3‐positive apoptotic cells than young counterparts (old Ki67: 4.6% ± 0.7%, young Ki67: 7.4% ± 1.0%, *p* = 0.03; old CC3: 1.5% ± 0.2%, young CC3: 0.4% ± 0.03%, *p* < 0.0001) (Figure 2D,E).

The extracellular matrix (ECM) is a critical component of the ovarian stroma, with hyaluronan and collagen being two major constituents (Amargant et al. 2020; Kinnear et al. 2020; Parkes et al. 2021). Using a hyaluronan binding protein assay and Picrosirius Red staining, we demonstrated that organoids produce both hyaluronan (young: 13.0% ± 1.7%, old: 10.2% ± 1.1%, *p* > 0.05) and collagen I & III (young: 2.8% ± 0.4%, old: 2.4% ± 0.3%, *p* > 0.05) (Figure 2F,G). Of note, organoids trended towards the age‐dependent decrease in hyaluronan that occurs in mouse and human ovaries (*p* = 0.147), but did not exhibit the characteristic age‐associated increase in collagen (Figure 2F,G) (Amargant et al. 2020; Briley et al. 2016; Landry et al. 2022; Umehara et al. 2022). To determine, however, if the organoids were responsive to a pro‐fibrotic stimulus, we treated them with transforming growth factor‐beta (TGF‐β), which has been demonstrated to induce collagen expression in several cell types including cardiac fibroblasts, alveolar fibroblasts, and myoblasts, as well as primary ovarian somatic cells cultured in a two‐dimensional monolayer (Amargant et al. 2025; Tsukui et al. 2020). Following TGF‐β treatment we assessed expression of *Col1a1* and *Acta2*, which encode one chain of collagen I and ⍺‐SMA, respectively, and are upregulated in fibrotic processes (Figure 2H,I) (Bochaton‐Piallat et al. 2016; Ding et al. 2021). TGF‐β treatment increased expression of *Col1a1* and *Acta2* in organoids generated from reproductively young (*Col1a1*: 2.8 ± 1.5‐fold increase, *p* = 0.03, *Acta2*: 23.1 ± 18.3‐fold increase, *p* = 0.03) and old (*Col1a1*: 3.0 ± 0.5‐fold increase, *p* = 0.01, *Acta2*: 2.4 ± 1.0‐fold increase, *p* > 0.05) mice, demonstrating the functional responsiveness of these organoids (Figure 2H,I).

Given that steroid hormone production is an important role of the ovary, we evaluated estradiol and progesterone production by ovarian somatic organoids at Days 1, 3, and 5 of culture to further probe their functional capacity (Figure 2J–K). Organoids generated from both young and old mice produced estradiol and progesterone (Figure 2J–K). Both organoids from young and old mice exhibited a significant increase in progesterone production between Day 1 (young: 6813.4 ± 832.2 pg/mL, old: 5370.5 ± 555.4 pg/mL) and Day 3 (young: 12232.2 ± 1380.6 pg/mL, *p* = 0.02, old: 10245.7 ± 908.6 pg/mL, *p* = 0.001) of culture (Figure 2J–K). However, while organoids from young mice demonstrated a significant increase in estradiol (Day 1: 17.3 ± 4.2 pg/mL, Day 5: 73.9 ± 12.2 pg/mL, *p* = 0.001) and progesterone (Day: 6813.4 ± 832.2 pg/mL, Day 5: 13507.9 ± 2829.3 pg/mL, *p* = 0.04) secretion between Days 1 and 5 of culture, organoids generated from old mice did not, indicative of impaired endocrine output as expected with age (Figure 2J–K) (Nelson et al. 1981).

### 2.3 Heterogeneous Cell Populations Are Maintained in Ovarian Somatic Organoids Irrespective of Age

**FIGURE 3:**
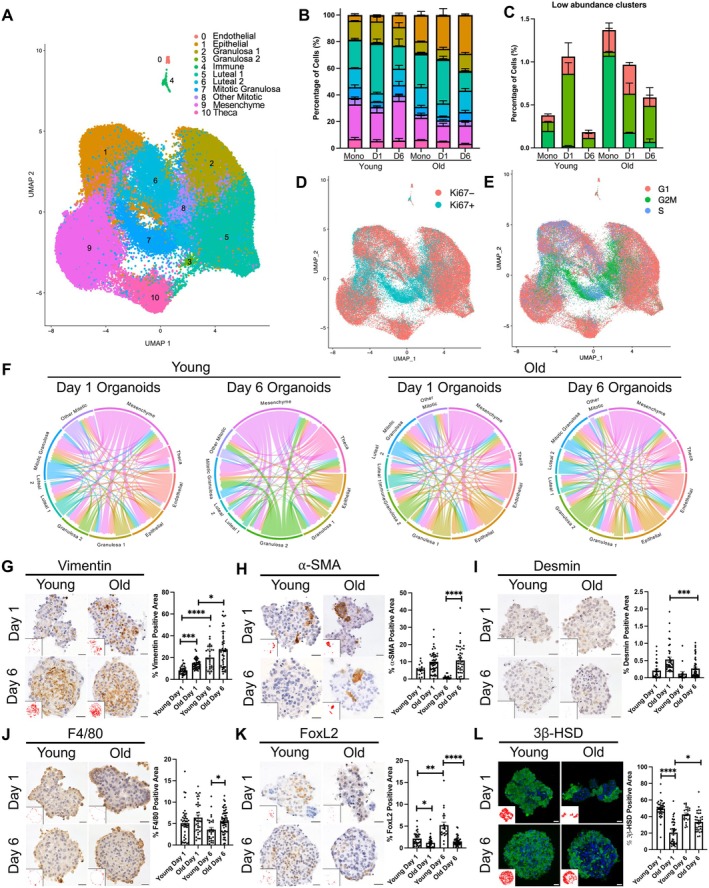
Heterogenous cell populations are maintained in ovarian somatic organoids irrespective of age, over 6 days in culture. (A) Uniform manifold approximation and projection (UMAP) plot of primary mouse ovarian somatic cells from reproductively young (6–12 weeks) and reproductively old (10–14 months) mice following plating in 2D, prior to organoid generation (monolayer) and following dissociation of ovarian somatic organoids at Days 1 and 6 of culture where scRNAseq was performed for two replicates per group and each biological replicate included cells pooled from at least five mice. Unbiased clustering revealed 11 distinct cell populations. (B, C) Quantification of the percentage of cells in each cluster for monolayer (Mono), Day 1 organoids (D1), and Day 6 (D6) organoids for each age. Error bars represent the standard error of the mean (SEM). (C) shows percentage of cells in low abundance clusters (0‐Endothelial, 3‐Granulosa 2, and 4‐Immune). Cluster color coding as in A. Statistical significance of differences in the percentage of cells in each cluster was not determined because scRNAseq was performed for two replicates per group. (D, E) UMAP plots showing cells colored based on (D) Ki67 expression (negative and positive) and (E) cell cycle stage (G1, G2M, S). (F) Chord diagrams showing cell–cell communication in Day 1 and 6 organoids for each age created using CellChat. The color of the chords indicate the cell cluster sending the signal and the size of the chords are proportional to signal strength. Statistical significance of differences in cell interactions was not determined because scRNAseq was performed on two replicates per group. (G–L) Representative images of vimentin (G, brown), alpha‐smooth muscle Actin (⍺‐SMA, H, brown), desmin (I, brown), F4/80 (J, brown), FOXL2 (K, brown), and 3β‐HSD (L, green) IHC staining of young and old ovarian somatic organoid sections at Days 1 and 6 of culture. For chromogenic staining nuclei were detected by hematoxylin (blue‐purple), and for fluorescent staining nuclei were detected by DAPI (blue). Scale bars = 20 μm. Insets are thresholded images highlighting positive pixels. Quantification of the positive area for each marker in young and old ovarian somatic organoids at Days 1 and 6 of culture (Young Day 1: *N* = 16–41, Old Day 1: *N* = 34–50, Young Day 6: *N* = 15–29, Old Day 6: *N* = 37–59). Error bars represent the standard error of the mean (SEM). *p < 0.05, **p < 0.01, ***p < 0.001, and ****p < 0.0001 by Kruskal–Wallis tests followed by Dunn's multiple comparisons tests. Statistical significance only for Young Day 1 versus Old Day 1, Young Day 6 versus Old Day 6, Young Day 1 versus Young Day 6, and Old Day 1 versus Old Day 6 is shown on graphs. All other statistically significant comparisons are listed in Table S2.

To obtain a more comprehensive understanding of the cell populations present within these ovarian somatic organoids throughout culture and determine whether any age‐dependent differences exist, we utilized single‐cell transcriptomic analysis. Single‐cell RNA‐sequencing (scRNAseq) was performed using primary ovarian somatic cells isolated from both reproductively young and old mice following two‐dimensional culture in vitro overnight in a differential plating step to remove oocytes but prior to organoid generation (monolayer), as well as from organoids generated from young and old mice on Day 1 and 6 of culture (Figure S4A). Unbiased clustering and uniform manifold approximation and projection (UMAP) analysis revealed 11 bioinformatically distinct cell clusters that were subsequently identified manually using known markers of mouse ovarian cell types (Figure 3A, Figure S4B–D) (Ben Yaakov et al. 2023; Isola et al. 2024; Morris et al. 2022). Luteal cells bioinformatically segregated into two clusters and were the most abundant cell type present (*n* = 27,269 cells), followed by mesenchymal cells (*n* = 15,235), and ovarian surface epithelial cells (OSE, *n* = 10,923) (Figure 3A). Other cell clusters included granulosa and theca cells, likely from the several different stages of follicles present in the ovary at any given time, as well as immune, endothelial, and mitotic cells (Figure 3A). The greater relative abundance of luteal, mesenchymal, and epithelial cells compared to granulosa and theca cells was consistent with our use of a stroma‐enriched fraction of the ovary. Importantly, these cell clusters were present in all organoids irrespective of age group and time in culture, demonstrating the ability of our organoid model to recapitulate and maintain the heterogeneous cellular composition of the ovarian stroma in vivo throughout culture in vitro (Figure 3B,C, Figure S4B,D). This finding was further supported by integration of our scRNAseq data with a published single‐cell atlas of the aging mouse ovary (Isola et al. 2024). Significant mixing of cells between datasets following integration and UMAP analysis suggests that organoids contain all somatic cell types present in the ovary in vivo (Figure S5A,B). To determine which cell populations contained proliferating cells, we visualized cells based on Ki67 expression (negative and positive) as well as their cell cycle stage‐associated transcriptomic signature (G1, G2M, S) (Figure 3D,E). Interestingly, mitotic cells were present in all cell clusters but there was an expected enrichment in the mitotic granulosa and other mitotic clusters whose specific cell type identity could not be assigned (Figure 3A,D,E). These results demonstrate the ability of our organoid model to support proliferation of all cell types and are consistent with the heterogeneity that is maintained even after 6 days of culture.

Nevertheless, the relative abundance of certain cell clusters shifted with age (Figure 3B,C, Figure S4B,D). For example, relative to young counterparts, primary ovarian somatic cells and organoids derived from reproductively old mice had a 3.2–5.5‐fold greater percentage of epithelial cells and a 4.5–6.4‐fold greater percentage of immune cells (Figure 3B,C, Figure S4B,D). However, immune cells in general were present in low numbers (Figure 3C, Figure S4B,D). Primary cells and organoids from old mice also contained smaller relative proportions of granulosa 1 cells (0.5–0.9‐fold change) and mesenchymal cells (0.5–0.6‐fold change) than those from young mice (Figure 3B, Figure S4B,D).

To determine which cell clusters exhibited the greatest age‐dependent changes in gene expression, we graphed the total number of differentially expressed genes (DEGs) with age per cluster for cells dissociated from organoids at Day 6 of culture (Figure S5C; Table S1). Epithelial (*n* = 959 DEGs), luteal 2 (*n* = 863 DEGs), and granulosa 1 (*n* = 597 DEGs) cells demonstrated the most significant age‐associated changes in gene expression (Figure S5C; Table S1). Gene Ontology analysis of genes differentially expressed in the epithelial cluster with age revealed downregulation of pathways associated with cell proliferation and bone‐related processes (Figure S5D). Pathways involved in response to oxidative stress and mitosis were downregulated with age in luteal 2 cells (Figure S5D). Pathways downregulated in granulosa 1 cells with age included those related to cell adhesion, cell migration, inflammatory response, as well as neuron‐associated pathways, which are commonly enriched in cumulus cells (Figure S5D) (Hernandez‐Gonzalez et al. 2006). Pathways related to aortic valve development, driven by Wnt‐signaling genes, were upregulated in granulosa 1 cells with age, and no pathways were significantly upregulated in epithelial or luteal 2 cells with age (Figure S5D).

CellChat analysis revealed cell communication and integration among the various cell types in ovarian somatic organoids. Consistent with previous studies of the aging mouse ovary in vivo, organoids from old mice had a greater number of intercellular interactions than young counterparts at both Day 1 (old: 3769 interactions, young: 3511 interactions) and Day 6 (old: 4477 interactions, young: 3233 interactions) of culture (Figure 3F) (Isola et al. 2024). At Day 1 of culture, the mesenchyme cluster had the greatest number of interactions in organoids derived from young mice, whereas the epithelial cluster had the greatest number of interactions in those derived from old mice (Figure 3F). However, mesenchymal cells were the most active in intercellular signaling in both organoids derived from reproductively young and old mice by Day 6 of culture (Figure 3F). Overall, cell–cell communication within organoids suggests that in addition to maintaining cellular heterogeneity, this culture method maintains cellular signaling capacity.

To further investigate age‐dependent differences in cell composition and organization of ovarian somatic organoids at the protein level, we performed immunohistochemistry using antibodies against the same markers of ovarian cell types as in Figure 1. Organoids contained fibroblasts, myofibroblasts, macrophages, and steroidogenic cells irrespective of age, but had age‐associated differences in the relative proportions of some cell populations (Figure 3G–L). Organoids generated from old mice exhibited a greater proportion of vimentin‐positive fibroblast‐lineage cells at Day 1 but not at Day 6 of culture compared to young counterparts (old Day 1: 14.3% ± 0.7%, young Day 1: 8.2% ± 0.6%, *p* = 0.0002; old Day 6: 27.4% ± 2.5%, young Day 6: 20.1% ± 2.1%, *p* > 0.05) (Figure 3G). Moreover, organoids from old mice contained significantly more ⍺‐SMA‐positive cells and F4/80‐positive macrophages than young counterparts at Day 6 of culture (old ⍺‐SMA: 10.8% ± 1.2%, young ⍺‐SMA: 1.1% ± 0.3%, *p* < 0.00001; old F4/80: 5.5% ± 0.3%, young F4/80: 3.6% ± 0.5%, *p* = 0.02) (Figure 3H,J). Although ⍺‐SMA marks both myofibroblasts and smooth muscle cells, organoids derived from both young and old mice at both Days 1 and 6 of culture only contained between 0.1% ± 0.1%–0.5% ± 0.1% of desmin‐positive smooth muscle cells, suggesting that ⍺‐SMA‐positive cells in ovarian somatic organoids are largely myofibroblasts (Figure 3I). FOXL2‐positive granulosa and granulosa‐lutein cells were less abundant in organoids from old mice at Day 1 and 6 of culture compared to those from young mice (old Day 1: 1.2% ± 0.2%, young Day 1: 2.1% ± 0.3%, *p* = 0.02; old Day 6: 1.5% ± 0.2%, young Day 6: 5.2% ± 0.7%, *p* < 0.0001) (Figure 3K). Similar to FOXL2‐positive cells, 3β‐HSD‐positive steroidogenic cells were also less abundant in organoids from old mice at Day 1, in comparison to organoids generated from young mice at Day 1, but were present in organoids from young and old mice in similar quantities by Day 6 (old Day 1: 20.6% ± 2.2%, young Day 1: 49.7% ± 1.7%, *p* < 0.0001; young Day 6: 42.6% ± 1.8%, old Day 6:33.5% ± 1.8%, *p* > 0.05) (Figure 3L). The spatial organization of macrophages and 3β‐HSD‐positive steroidogenic cells in organoids generated from old mice was similar to young counterparts in that macrophages were enriched in the perimeters of organoids and 3β‐HSD‐positive cells were depleted from the cores (Figure 3J,L).

### 2.4 Age‐Dependent Changes in Actin and Cell Adhesion Pathways in Ovarian Somatic Cells Are Suggestive of Increased Cellular Stiffness

**FIGURE 4:**
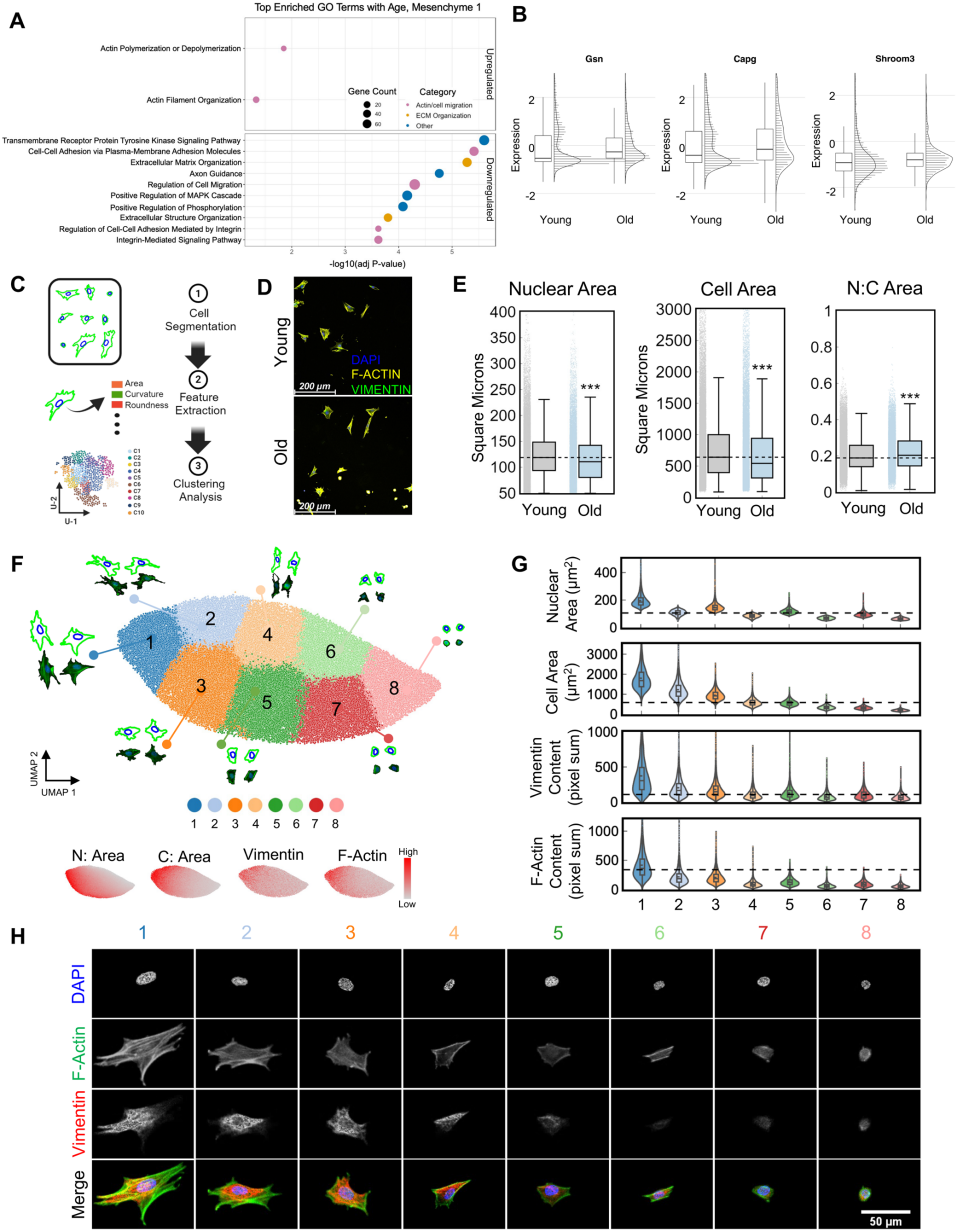
Actin pathways, which regulate cell morphology, are upregulated in ovarian mesenchyme 1 cells with age. (A) GO analysis of differentially expressed genes in the Mesenchyme 1 cluster with age for monolayer cells. Pathways upregulated and downregulated with age are labeled and displayed in manually grouped categories. scRNAseq was performed for two replicates per age and each biological replicate included cells pooled from at least five mice. (B) Raincloud plots showing expression of genes driving upregulated Actin‐related pathways in the Mesenchyme 1 cells from young and old mice. (C) Schematic of the morphological analysis pipeline. Over 89,000 cells spanning both ages (young and old) and all treatments (vehicle, LatA, and JASP) collected from 8 independent biological replicates were utilized for morphological analysis in all subsequent panels in Figure 4. (D) Representative images of ovarian somatic cells from reproductively young (6–12 weeks) and reproductively old (10–14 months) mice. F‐Actin (phalloidin, yellow), vimentin (green), and DAPI (blue) were detected by immunocytochemistry. Scale bars = 200 μm. (E) Box and whisker plots showing quantification of nuclear area, cellular area, and nuclear to cellular area ratio for ovarian somatic cells from young and old mice. ***p < 0.001 by *t*‐tests. (F) 2D UMAP visualization based on 106 morphological parameters. 8 k‐means clusters are layered on top of the UMAP space with representative cellular (outer, green) and nuclear (inner, blue), and raw image morphology for each cluster. Panels below show distribution of high (red) and low (gray) nuclear area (*N*: Area), cellular area (C: Area), vimentin expression, and F‐Actin expression displayed within the UMAP space. High and low represent standard scaling of expression across the dataset. (G) Violin plots of the nuclear area, cellular area, vimentin, and F‐Actin content for each of the 8 k‐means clusters. (H) Representative images of an ovarian somatic cells within each morphological cluster. DAPI (blue), F‐Actin (phalloidin, green), and vimentin (red) were detected by immunocytochemistry. Brightness of fluorescent staining was adjusted equivalently for all images to highlight localization. Scale bar = 50 μm.

To identify age‐related changes in the ovarian somatic compartment at the cellular level, which may underlie altered organoid formation and function, we examined the gene expression patterns of primary ovarian somatic cells from reproductively young and old mice which were used as the input to generate the organoids (monolayer) separately (Figure S6A). This analysis revealed two additional clusters that were not present in the UMAP when cells dissociated from organoids were incorporated in the analysis (Figure 3A, Figure S6A,B) (Ben Yaakov et al. 2023; Isola et al. 2024; Morris et al. 2022). In the analysis of the monolayer alone, the mesenchymal cells segregated into two clusters, and myeloid cells and lymphocytes separated into distinct clusters (Figure S6A). To define the identity of cells present in each of the mesenchyme clusters we performed Gene Ontology analysis of genes differentially expressed within each cluster. High expression of *Col1a2* and enrichment of biological processes and cellular components related to ECM function in the mesenchyme 1 cluster suggested that cells in this cluster were matrix fibroblasts (Figure S6A–C) (Landry et al. 2022). Interestingly, pathways associated with the actin cytoskeleton were also enriched in this cluster (Figure S6C). To evaluate age‐dependent changes in the mesenchyme 1 cluster, we performed Gene Ontology analysis of genes differentially expressed in this cluster with advanced reproductive age (Figure 4A, Table S3). Actin polymerization or depolymerization and actin filament organization pathways were upregulated with age, driven by increased expression of genes that play roles in actin binding, actin nucleation, actin capping, as well as adherens and tight junctions (Figure 4A,B, Table 1). Cell adhesion and cell migration pathways were downregulated with age (Figure 4A). Downregulation of cell adhesion‐related pathways was driven by decreased expression of claudins, cadherins, and integrins, among other genes (Table 2). In general, the cytoskeleton, particularly cytoskeletal components F‐actin and intermediate filaments, is considered to be a primary contributor to cytoplasmic stiffness, and actin filament assembly has been correlated with a rapid rise in cytoplasmic stiffness (Lee et al. 2006; Wirtz 2009). Increased cellular stiffness, driven by increased F‐actin content and organization, could contribute to decreased cell–cell adhesion (Phillip et al. 2015). Of note, increased cellular stiffness is a hallmark of aging cells in other tissues, but to date has not been examined in the context of ovarian aging (Phillip et al. 2015).

**TABLE 1:** Genes driving upregulated Actin pathways in mesenchyme 1 cells from reproductively old mice.

Gene symbol	Gene name	Average log_2_fold‐change	Adjusted *p*‐value	Function of encoded protein^a^
Lcp1	Lymphocyte cytosolic protein 1	13.158772	3.05E‐73	Enables Actin filament binding activity; Acts in Actin filament bundle assembly
Gsn	Gelsolin	10.3239145	1.57E‐25	Actin binding protein; Involved in Actin filament capping; Involved in Actin polymerization and depolymerization
Was	Wiskott‐Aldrich syndrome	5.30194898	2.99E‐26	Actin signaling; Actin nucleation and assembly
Shroom3	Shroom family member 3	3.44015737	7.47E‐10	Enables Actin binding activity
Capg	Capping Actin protein, gelsolin like	3.06743619	0.00053986	Caps barbed ends of Actin filaments
Avil	Advillin	1.26971477	0.00056432	Enables Actin filament binding activity
Pstpip2	Proline‐serine–threonine phosphatase‐interacting protein 2	1.1908554	5.52E‐17	Enables Actin filament binding activity; Involved in Actin filament polymerization
Aif1	Allograft inflammatory factor 1	0.87640441	1.40E‐22	Enables Actin filament binding activity
Cgnl1	Cingulin‐like 1	0.67256897	1.84E‐10	Mediates adherens and tight junction assembly and maintenance

^a^ Functions of encoded proteins were sourced from the NCBI Gene Database (Sayers et al. 2022).

**TABLE 2:** Top 20 genes greatest absolute Log_2_fold‐change driving downregulated cell adhesion pathways in mesenchyme 1 cells from reproductively old mice.

Gene symbol	Gene name	Average log_2_fold‐Change	Adjusted *p*‐value	Function of encoded protein^a^
Cdh10	Cadherin 10	−14.771257	2.86E‐05	Mediates calcium‐dependent cell–cell adhesion
Il1rapl1	Interleukin 1 receptor accessory protein‐like 1	−13.845918	2.80E‐54	Involved in heterophilic cell–cell adhesion via plasma membrane cell adhesion molecules
Cldn10	Claudin 10	−13.597923	9.59E‐117	Integral membrane protein; Component of tight junction strands
Itgb4	Integrin beta 4	−13.50424	2.51E‐33	Mediates cell‐matrix or cell–cell adhesion; Laminin receptor
Tenm2	Teneurin transmembrane protein 2	−11.642849	1.11E‐42	Enables cell adhesion molecule binding activity; Involved in heterophilic cell–cell adhesion via plasma membrane cell adhesion molecules
Dpp4	Dipeptidylpeptidase 4	−11.509687	4.43E‐09	Cell adhesion; Glucose and Insulin metabolism; Immune regulation
Skap1	Src family associated phosphoprotein 1	−10.43278	4.55E‐20	Adhesion and degranulation‐promoting T cell adaptor protein; Positive regulation of cell adhesion and protein localization to plasma membrane
Prkd1	Protein kinase D1	−10.346048	1.84E‐53	Regulation of cell shape and adhesion
Cdh15	Cadherin 15	−9.959104	2.21E‐08	Calcium‐dependent intercellular adhesion glycoprotein
Pecam1	Platelet/endothelial cell adhesion molecule 1	−9.8910307	4.83E‐12	Involved in integrin activation, angiogenesis, leukocyte migration
Cd84	CD84 antigen	−9.6961405	2.56E‐89	Homophilic adhesion molecule
Cdh4	Cadherin 4	−9.6008396	7.93E‐33	Calcium‐dependent cell–cell adhesion glycoprotein
Ptprt	Protein tyrosine phosphatase receptor type T	−9.350224	1.07E‐67	Cellular adhesion and signal transduction
Mpzl2	Myelin protein zero‐like 2	−9.2835348	1.14E‐12	Mediates cell adhesion through homophilic interaction
Cldn5	Claudin 5	−8.8057337	4.30E‐11	Integral membrane protein; Component of tight junction strands
Ccl5	C‐C motif chemokine ligand 5	−7.9391496	1.78E‐54	Chemoattractant
Cd3e	CD3 antigen, epsilon polypeptide	−7.7190168	1.41E‐07	Component of the T‐cell receptor‐CD3 complex
Cdh12	Cadherin 12	−6.7639572	1.49E‐46	Mediates calcium‐dependent cell–cell adhesion
Cdh13	Cadherin 13	−6.7073008	4.91E‐07	Mediates calcium‐dependent cell–cell adhesion; Implicated in modulation of angiogenesis
Plek	Pleckstrin	−6.5114596	2.42E‐54	Involved in Actin cytoskeleton organization

^a^ Functions of encoded proteins were sourced from the NCBI Gene Database (Sayers et al. 2022).

### 2.5 Ovarian Somatic Cells Have Heterogenous Morphology That Is Altered With Advanced Reproductive Age

In addition to impacting cellular stiffness, the actin cytoskeleton plays a central role in regulating cell morphology (Chalut and Paluch 2016; Pollard and Cooper 2009). Cell morphology has been linked to biological properties of cells including cell cycle progression, metastatic potential, as well as gene expression (Nguyen et al. 2016; Wu et al. 2020; Yin et al. 2013). Importantly, cell morphology encodes aging information and can be used as a predictive biomarker of cellular age (Phillip et al. 2017). To evaluate the morphology of ovarian somatic cells isolated from reproductively young and old mice, we employed a previously established high throughput cellular phenotyping scheme which extracts hundreds of parameters describing the morphology of cells and their corresponding nuclei (Wu et al. 2015; Wu et al. 2020). These morphological parameters include metrics that describe both the size and shape of cells and nuclei, thereby allowing direct interrogation and comparison of cell morphology at the single‐cell level (Wu et al. 2015, 2020). To this end, primary ovarian somatic cells isolated from young and old mice were seeded onto collagen I‐coated glass substrates, fixed, and stained to visualize F‐actin, vimentin, and DNA (Figure 4C,D). Individual cells were segmented using CellProfiler, and DAPI and F‐actin staining was used to determine nuclear and cellular borders, respectively. Vimentin staining was performed to identify fibroblasts and myofibroblasts, which demonstrated an age‐dependent enrichment in pathways associated with the actin cytoskeleton (Figure 4A). From the nuclear and cellular boundaries, we computed 228 morphological parameters along with the corresponding F‐actin and vimentin expression per cell (Figure 4C) (Wu et al. 2015, 2020). These 228 parameters were subsequently reduced to 106 following orthogonality analysis to identify the parameters that best captured the variance within the dataset. Using these 106 parameters, we performed clustering analysis of more than 89,000 single cells collected from eight biological replicates (Figure 4C).

**FIGURE 5:**
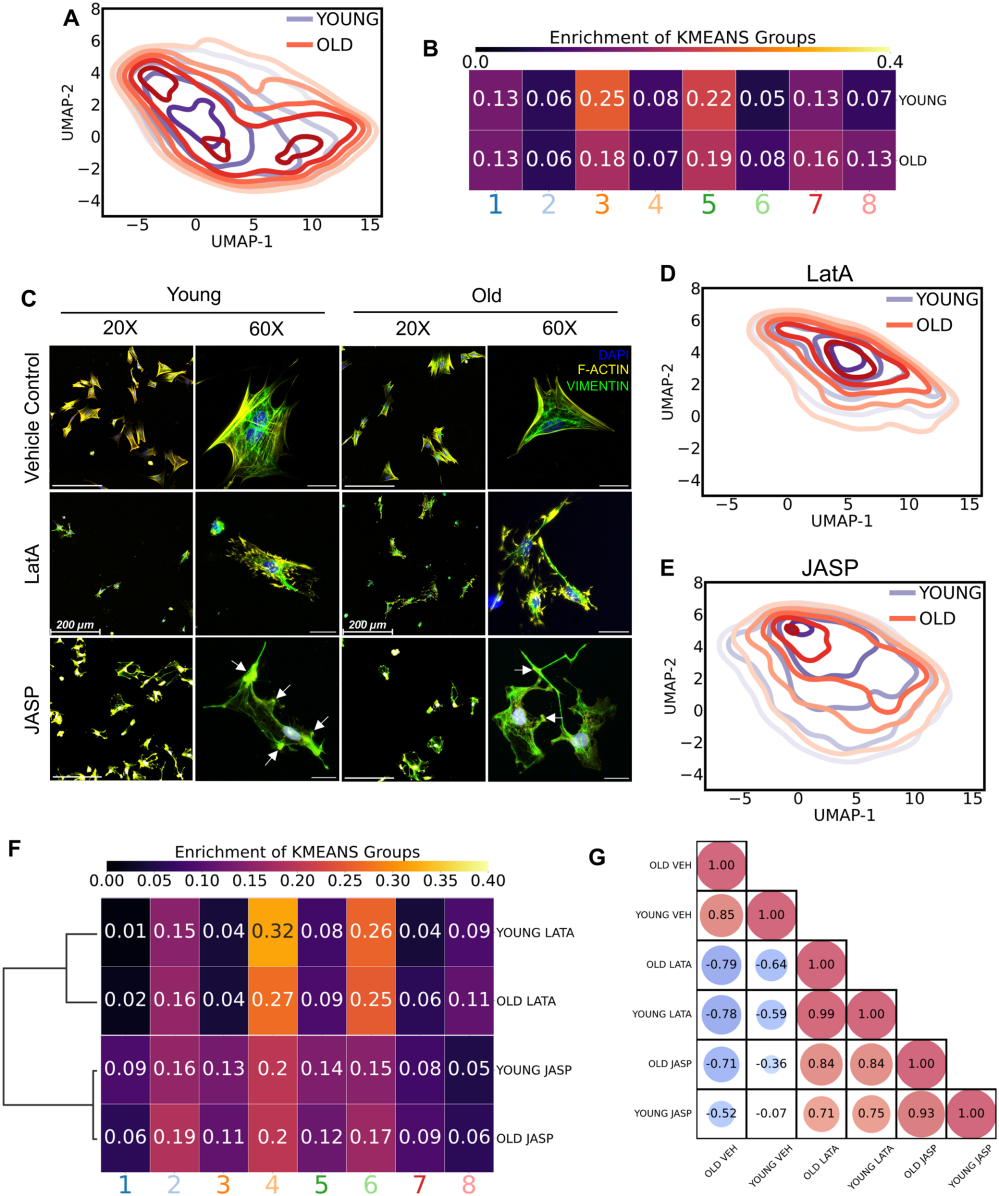
Ovarian somatic cell morphology shifts with age and upon modulation of the Actin cytoskeleton. (A) Contour overlays on the UMAP space showing enriched morphology for ovarian somatic cells from young (purple) and old (red) mice. Over 89,000 cells spanning both ages (young and old) and all treatments (vehicle, LatA, and JASP) collected from 8 independent biological replicates were utilized for morphological analysis for this and all subsequent panels in Figure 5. (B) Heatmap showing the fractional abundance of ovarian somatic cells from young and old mice within each k‐means cluster. (C) Representative 20× and 60× images of ovarian somatic cells from young and old mice treated with vehicle control, Latrunculin A (LatA), and Jasplakinolide (JASP). F‐Actin (phalloidin, yellow), vimentin (green), and nuclei (DAPI, blue) were detected by immunocytochemistry. Arrows show distinct Actin enrichments. 20× Scale bars = 200 μm. 60× Scale bars = 50 μm. (D) Contour overlays on the UMAP space showing enriched morphology for ovarian somatic cells from young (purple) and old (red) mice treated with LatA. (E) Contour overlays on the UMAP space showing enriched morphology for ovarian somatic cells from young (purple) and old (red) mice treated with JASP. (F) Heatmap showing the fractional abundance of ovarian somatic cells from young and old mice with or without LatA or JASP treatment within each k‐means cluster. (G) Bubble plot showing correlation of morphological enrichment between experimental groups.

On average, ovarian somatic cells isolated from reproductively old mice had smaller nuclear and cellular area compared to young counterparts, resulting in a significant age‐associated increase in the nuclear‐to‐cellular area ratio (Figure 4E). To gain deeper insights into the morphological differences, we performed clustering and dimensional reduction analyses of the cell and nuclear morphology across conditions to identify groups of cells with similar morphology. K‐means clustering analysis visualized using 2D UMAP identified 8 morphological clusters of primary ovarian somatic cells, each with distinct cellular and nuclear morphology (Figure 4F,H). Cluster 1 corresponded to cells with the largest nuclear and cellular area, and highest F‐actin and vimentin staining, suggesting a fibroblast identity (Figure 4F–H). Cluster 8, on the other hand, corresponded to the smallest and roundest cells with low F‐actin and vimentin expression, suggestive of an epithelial identity (Figure 4F–H). However, there was a distribution and not every cell in Cluster 1 had a larger nuclear and cellular area or greater F‐actin and vimentin content than cells in any other cluster (Figure 4G). Similarly, not every cell in Cluster 8 was smaller or had less F‐actin and vimentin staining than cells in other clusters (Figure 4G). In general, primary ovarian somatic cells isolated from old mice were enriched in cluster 8 compared to young counterparts (Figure 5A,B). The age‐associated enrichment in this cluster is consistent with the increased abundance of ovarian epithelial cells with age (Figure 3B, Figure S4B,D) (Bajwa et al. 2016; Mara et al. 2020). Moreover, while cells from both young and old mice exhibited similar abundance of clusters 1, 2, 4, 6, and 7, cells from young mice were enriched in clusters 3 and 5 (Figure 5A,B). Interestingly, although the enriched morphological clusters were relatively consistent between different groups of young mice, ovarian somatic cell morphology was highly heterogeneous among different groups of old mice (Figure S7A). Taken together, we show that ovarian somatic cells have heterogeneous morphology. Moreover, the relative abundance of various cell morphologies shifts with age, further demonstrating altered properties of the aging ovarian somatic compartment at a cellular level.

### 2.6 Modulating the Actin Cytoskeleton Shifts Ovarian Somatic Cell Morphology and Alters the Aggregation of Ovarian Somatic Organoids

Our results demonstrate an age‐related upregulation of actin pathways in the mesenchyme 1 cluster and an age‐dependent decrease in cell adhesion pathways, which is indicative of increased cellular stiffness (Figure 4A). Given the high actin content in cluster 1, we hypothesized that cells in this cluster may contribute to the age‐dependent increase in cellular stiffness and that the morphology of these cells may be responsive to actin depolymerization. To directly evaluate this, we treated primary cells isolated from reproductively young and old mice with 1 μM Latrunculin A (LatA) for 45 min, which depolymerizes actin filaments and sequesters actin monomers to prevent polymerization (Fujiwara et al. 2018). Treatment with LatA successfully disrupted the actin cytoskeleton in primary ovarian somatic cells isolated from reproductively young and old mice (Figure 5C). Moreover, LatA treatment significantly depleted the abundance of cells in clusters 1 and 3, in addition to normalizing the age‐associated enrichment in cluster 8 (Figure 5B,D,F). Additionally, LatA treatment shifted cell morphology in both young and old mice from clusters 1, 3, and 5 to clusters 4 and 6, which are comparatively smaller cells with smaller nuclear area and reduced F‐actin and vimentin content (Figure 4F–H, Figure 5B,D,F). Furthermore, ovarian somatic cells treated with Lat A exhibited homogeneous morphology irrespective of the age of the mice from which they were isolated (Figure 5D,F). This homogeneity was evidenced by a correlation value of 0.99 when comparing the morphological enrichment of cells derived from reproductively young and old mice following LatA treatment, compared to a correlation value of 0.83 when comparing vehicle‐treated controls (Figure 5G). To determine whether LatA treatment had an equal impact on the morphology of ovarian somatic cells isolated from reproductively young and old mice, we plotted the distance of each untreated cell to the centroid of all the LatA‐treated cells for each age in the UMAP space (Figure S8A). LatA had a greater effect on the morphology of ovarian somatic cells from old mice compared to young counterparts as quantified by a greater distance of each old cell at baseline to the centroid of the UMAP distribution following LatA treatment (Figure S8A).

To further investigate the impact of modulation of the actin cytoskeleton on cell morphology, we treated ovarian somatic cells isolated from reproductively young and old mice with 1 μM Jasplakinolide (JASP) for 45 min, which promotes actin polymerization and stabilizes actin filaments (Schulze et al. 2012). JASP treatment stabilized actin, resulting in the appearance of distinct actin enrichments in ovarian somatic cells isolated from reproductively young and old mice (Figure 5C). Treatment with JASP shifted cell morphology in both young and old mice from clusters 3 and 5 to clusters 2, 4, and 6, such that all clusters 2–6 had similar enrichment (Figure 5B,E,F). Thus, JASP treatment resulted in more heterogeneous cell morphology than LatA treatment. Ovarian somatic cells isolated from reproductively young and old mice treated with JASP had a correlation value of 0.93 indicating they exhibited similar morphology following JASP treatment, irrespective of age (Figure 5G). However, centroid plots demonstrate that JASP treatment had a greater effect on the morphology of ovarian somatic cells from old mice than young counterparts (Figure S8B).

**FIGURE 6:**
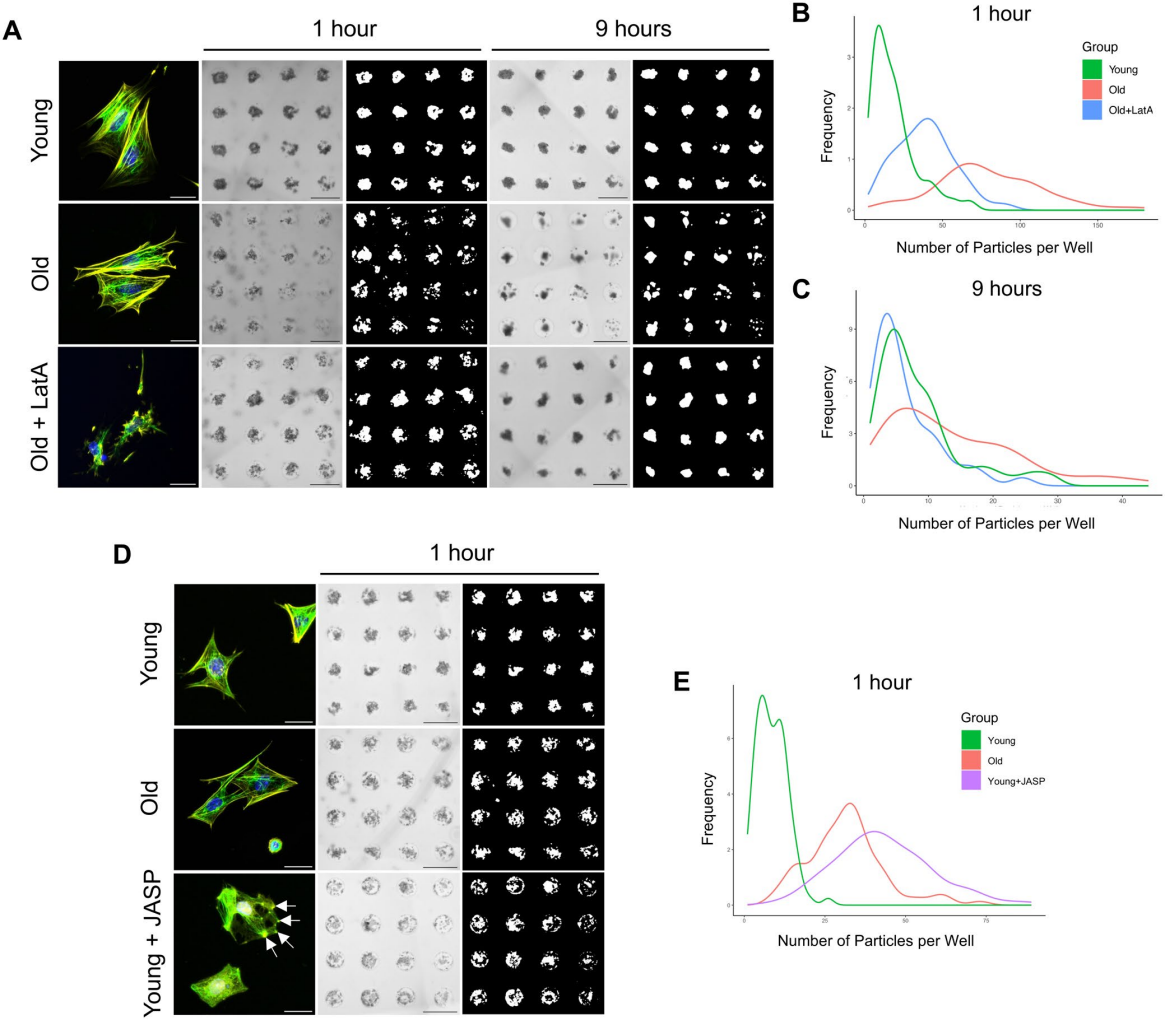
Modulating the Actin cytoskeleton alters the aggregation of ovarian somatic organoids. (A) Representative images of primary ovarian somatic cells following treatment with Latrunculin A (LatA) or vehicle control. F‐actin (phalloidin, yellow), vimentin (green), and nuclei (DAPI, blue) were detected by immunocytochemistry. Scale bars = 50 μm. Representative images of organoids generated from young and old primary ovarian somatic cells following treatment with Latrunculin A (LatA) or vehicle control at 1 and 9 h post seeding into agarose micromolds. Scale bars = 500 μm. Right panels for each timepoint are thresholded masks of cells in agarose micromolds. (B, C) Kernel‐smoothed distribution curves for histograms quantifying the frequency of different numbers of particles per well at 1 h (B) and 9 h (C) following seeding of primary ovarian somatic cells from young (green) and old (red) mice treated with a vehicle control or primary ovarian somatic cells from old mice treated with LatA (blue) into agarose micromolds. *N* = 1 micromolds per condition. Representative images for *N* = 2 additional micromolds per condition are shown in Figure S9A,B. (D) Representative images of primary ovarian somatic cells following treatment with Jasplakinolide (JASP) or vehicle control. F‐actin (phalloidin, yellow), vimentin (green), and nuclei (DAPI, blue) were detected by immunocytochemistry. Arrows show distinct actin enrichments. Scale bars = 50 μm. Representative images of organoids generated from young and old primary ovarian somatic cells following treatment with Jasplakinolide (JASP) or vehicle control at 1 h post seeding into agarose micromolds. Scale bars = 500 μm. Right panels for each timepoint are thresholded masks of cells in agarose micromolds. (E) Kernel‐smoothed distribution curves for histograms quantifying the frequency of different numbers of particles per well at 1 h following seeding of primary ovarian somatic cells from young (green) and old (red) mice treated with a vehicle control or primary ovarian somatic cells from young mice treated with JASP (purple) into agarose micromolds. *N* = 1 micromolds per condition. Representative images for *N* = 3 additional micromolds per condition are shown in Figure S9F–H.

Increased cellular stiffness may underlie the poor ability of ovarian somatic cells derived from old mice to aggregate into fully functional organoids (Figure 2A) (Phillip et al. 2015). To evaluate the functional role of actin polymerization and cellular stiffness on age‐dependent organoid formation, we tested whether partial depolymerization of the actin cytoskeleton through a short‐term treatment with LatA could rescue the age‐dependent impairment in organoid formation. Organoids generated using ovarian somatic cells from reproductively young mice served as a baseline control. Interestingly, LatA treatment of ovarian somatic cells derived from reproductively old mice resulted in enhanced organoid formation at 1 and 9 h post cell seeding relative to vehicle treated controls (Figure 6A; Figure S9A,B). In fact, quantitative analysis of the frequency of the number of particles per microwell demonstrated that LatA treatment of ovarian somatic cells isolated from reproductively old mice resulted in an organoid aggregation profile that more closely resembled that of young mice (Figure 6B,C). To determine if the increased aggregation potential of LatA‐treated ovarian somatic cells isolated from old mice was associated with improved functional capacity, we cultured organoids for 6 days following LatA treatment of ovarian somatic cells and evaluated cell proliferation and apoptosis by immunohistochemistry. LatA‐treated organoids generated from old mice contained a higher percentage of Ki67‐positive area (4.6% ± 0.6%) compared to untreated young (2.0% ± 0.2%, *p* < 0.05) and old controls (1.1% ± 0.2%, *p* < 0.0001) (Figure S9C,D). Moreover, LatA treatment partially rescued the age‐dependent increase in apoptotic cells with 0.1% ± 0.02% CC3‐positive area in LatA‐treated organoids generated from old mice compared to 0.3% ± 0.04% in untreated old controls (*p* < 0.0001) (Figure S9C,E). To further understand the role of the actin cytoskeleton in organoid formation, we assessed aggregation potential of ovarian somatic cells from reproductively young mice following short‐term treatment with the actin polymerizing and stabilizing agent, JASP. JASP treatment reduced the aggregation of ovarian somatic cells from young mice at 1 h post cell seeding compared to vehicle treated controls such that they more closely resembled the behavior of ovarian somatic cells isolated from reproductively old mice (Figure 6D,E, Figure S9F–H). Overall, age‐associated changes to the ovarian somatic compartment at a cellular level underlie differences in the capacity for aggregation.

## 3 Discussion

In this study, we developed a robust organoid model of the mouse ovarian somatic compartment. This represents an advance in the field in that it preserves the cellular heterogeneity of the native tissue, demonstrates organ‐specific functionality, and provides insights into mechanisms of ovarian aging. Ovarian somatic organoids were solid structures with proliferative cells present throughout and apoptotic cells restricted to the perimeter. They contained key ovarian cell types, including fibroblasts, epithelial, endothelial, immune, granulosa, theca, and luteal cells, and self‐organized such that 3β‐HSD‐positive steroidogenic cells were consistently depleted from the core and macrophages were enriched at the perimeter. Additionally, organoids produced an extracellular matrix, were responsive to exogenous stimuli, and secreted hormones. Interestingly, organoids generated from reproductively old mice demonstrated impaired aggregation and decreased growth compared to young counterparts. Organoids exhibited age‐dependent changes in relative cell composition and functional output with respect to endocrine function. At a cellular level, aging was associated with upregulation of pathways involved in actin dynamics in ovarian mesenchymal cells, as well as downregulation of pathways involved in cell adhesion. Enrichment of these pathways suggested increased cellular stiffness with age. Cell morphology, which is regulated by the actin cytoskeleton, was heterogeneous among ovarian somatic cells and was altered with age. Modulation of actin with Latrunculin A or Jasplakinolide shifted ovarian somatic cell morphology. LatA treatment improved aggregation, increased cell proliferation, and reduced apoptosis in organoids from old mice. Jasplakinolide treatment reduced aggregation potential of ovarian somatic cells from young mice. Overall, insights gained from this model suggest that aging of the ovarian somatic compartment may have cellular origins.

One of the greatest limitations of current ex vivo models of the ovarian somatic compartment is their inability to recapitulate and maintain cellular heterogeneity. Attempts at culturing ovarian somatic cells in 2D result in loss of heterogeneity over time in culture, and existing organoid models, including ovarian surface epithelial organoids, only capture a single cell type (Kwong et al. 2009; Tingen et al. 2011). In contrast, we have demonstrated the maintenance of fibroblasts, epithelial, immune, and steroidogenic cell populations within organoids over 6 days in culture, by which point 2D monolayer cultures of ovarian somatic cells are largely overtaken by macrophages (Tingen et al. 2011). Future studies are ongoing to interrogate how extended time in culture impacts ovarian somatic organoid structure and function. Moreover, we have demonstrated the functionality of these cell populations in their ability to respond to a pro‐fibrotic stimulus as well as their ability to produce hormones. This fidelity in composition and function will allow use of this organoid model to study interactions between the ovarian stroma and other cells and tissues. Importantly, we demonstrate that oocytes and intact follicles are not needed to assemble and organize the ovarian stroma, but future studies may use ovarian somatic organoids to understand the impact of oocyte‐secreted factors on the stroma and vice versa, particularly in the context of aging and the corresponding decline in oocyte quality and quantity (Broekmans et al. 2009). Ovarian somatic organoids may also provide a controlled model system for future studies to interrogate the signals required to assemble follicles upon the incorporation of oocytes, especially given that unlike other in vitro culture methods that result in rapid luteinization, granulosa cells are maintained within organoids over 6 days of culture, as we demonstrate through scRNAseq analysis, and are likely responsible for the estradiol secretion observed throughout culture (Murphy 2000). Furthermore, these organoids can be generated using rhesus macaque ovarian tissue, but additional studies with a larger sample size are required to more fully characterize nonhuman primate ovarian somatic organoid composition and function. In addition to applying this model to other species, these methods may also be used to generate organoids to probe the contribution of the ovarian stroma in conditions such as polycystic ovary syndrome, ovarian cancer, and post‐chemotherapy or radiation.

Interestingly, IntestiCult mouse intestinal organoid growth medium best supported ovarian somatic organoid aggregation and growth while maintaining key ovarian cell types over 6 days of culture. Although IntestiCult is a proprietary formula and the composition is unknown, other media that have been used to establish and culture intestinal organoids contain factors involved in Wnt‐signaling, including Wnt3A and Rspo1 (Pleguezuelos‐Manzano et al. 2020). The Wnt‐signaling pathway plays a role in organoid formation in other cell types (Lehmann et al. 2020; Nalapareddy et al. 2017; Pentinmikko et al. 2019; Uchida et al. 2019). Future studies are needed to identify the specific components of that promote ovarian organoid formation and support proliferation while maintaining cellular heterogeneity.

Ovarian somatic organoids exhibited several age‐dependent changes, including differences in relative cell composition. Organoids generated from reproductively old mice had a larger proportion of epithelial cells than young counterparts, which may be due to ovarian surface epithelial hyperplasia that occurs with age (Auersperg et al. 2001; Bajwa et al. 2016; Isola et al. 2024; Mara et al. 2020; Thung et al. 1956). Consistent with the age‐associated increase in epithelial cells, ovarian somatic cells from reproductively old mice were enriched for a population of cells with small, rounded morphology and low vimentin expression, suggesting epithelial identity. Additionally, although present at low abundance, organoids from old mice had an increased proportion of immune cells, reflective of the inflammatory environment of the aging ovary (Briley et al. 2016; Isola et al. 2024; Lliberos et al. 2021). Moreover, organoids from old mice contained fewer steroidogenic cells, especially granulosa cells, which is consistent with age‐dependent depletion of ovarian follicles (Faddy et al. 1976; Isola et al. 2024). Correspondingly, organoids from old mice exhibited blunted hormone production compared to organoids from young mice. This attenuated hormone secretion could be due to the smaller number of steroidogenic cells in addition to reduced functionality of the remaining cells, given the downregulation of pathways related to oxidative stress response in luteal 2 cells with age. In addition to granulosa, theca, and luteal cells, previous studies have identified interstitial cells in the ovarian stroma as another site of steroidogenesis (Kinnear et al. 2020; Makita and Hirose 1994). Thus, future studies are needed to determine which cell populations in ovarian somatic organoids are responsible for steroid hormone production and to further characterize how these cell populations change with age. Although we did not control for the estrous cycle in our approach, age is likely the primary driver impacting the observed changes in cell composition and behavior in the organoids. Previously published scRNAseq data of the aging mouse ovary demonstrate age‐associated changes in gene expression are more robust than those due to differences in estrous cycle stage as samples cluster by age regardless of bioinformatically determined cycle stage (Isola et al. 2024).

Organoids did not recapitulate age‐related changes to the extracellular matrix observed in vivo, particularly the age‐dependent increase in collagen and decrease in hyaluronan (Amargant et al. 2020; Briley et al. 2016). Myofibroblasts are the primary cell type responsible for ECM secretion during wound healing and fibrosis (Briley et al. 2016; Klingberg et al. 2013). Thus, the inability of organoids to establish the age‐related increase in collagen content present in the ovary in vivo may be due, in part, to the similar percentage of myofibroblasts in organoids derived from both young and old mice at Day 1 of culture. It is possible that the procedure of isolating primary ovarian somatic cells from reproductively old mice selects for the healthiest cells. It is also possible that the age‐associated fibrosis in the ovary makes it harder to isolate particular cell populations. For example, isolation of hepatic stellate cells, the main fibrogenic cell type in the liver, is more difficult from fibrotic tissue relative to normal tissue (Mederacke et al. 2015). Therefore, specialized protocols may be required to isolate activated myofibroblasts from reproductively old ovaries. In addition, further studies are needed to determine whether specific ovarian cell types from reproductively old mice maintain aging phenotypes in vitro or whether they are reprogrammed. Interestingly, organoids from old mice contained a significantly higher proportion of myofibroblasts by Day 6 of culture, suggesting that age‐related changes may in fact re‐establish in vitro. Given the time required for matrix deposition and remodeling, a longer time in culture may be needed to observe age‐associated changes in the ECM. In addition, ovarian somatic organoids do not contain oocytes, and thus they are an inherent model of ovarian aging given the well documented age‐dependent decrease in gamete quantity (Broekmans et al. 2009). Thus, we anticipate that the loss of oocytes may promote aging in the ovarian somatic compartment with longer term cultures. Nevertheless, several robust age‐dependent differences are captured in the organoid model, including the striking contrast in aggregation potential of organoids from reproductively young and old mice, which may be a result of an increase in cellular stiffness with advanced reproductive age.

In fact, similar to ovarian somatic organoids, an age‐associated reduction in organoid formation has previously been reported for human and mouse intestinal epithelial cells, mouse alveolar epithelial type II cells, as well as human tendon stem/progenitor cells, suggesting that this phenotype is not an organ‐specific pattern, but rather a common feature of aging in a dish (Sun et al. 2023; Torrens‐Mas et al. 2021). In addition to a reduction in the overall capacity to form organoids, intestinal epithelia from aged mice formed smaller organoids with fewer *de novo* crypts and lower cell numbers (Pentinmikko et al. 2019; Uchida et al. 2019). The age‐dependent decrease in organoid formation of intestinal epithelial cells and alveolar cells has been correlated to increased cellular senescence, reduced expression of tissue‐specific stemness markers, as well as perturbations to mTOR and Wnt signaling with age (He et al. 2020; Lehmann et al. 2020; Nalapareddy et al. 2017; Pentinmikko et al. 2019; Uchida et al. 2019). Future studies are required to determine if perturbed mTOR or Wnt signaling underlie the age‐dependent decrease in aggregation of ovarian somatic cells and if treatment with geroprotectors improves ovarian somatic organoid formation. Moreover, using an established cell sheet model for in vitro tenogenesis, previous studies determined that human tendon stem/progenitor cells from aged donors required more time to form a cell sheet than young counterparts (Yan et al. 2020). Interestingly, aging was associated with worse cell elongation and cytoskeletal organization in tendon cell sheets, as well as accumulation of actin stress fibers (Kohler et al. 2013; Yan et al. 2020). These data suggest that perturbations to the actin cytoskeleton may contribute to the age‐dependent decrease in organoid formation across multiple tissues.

In the context of ovarian aging, “stiffness” has typically referred to altered biomechanical properties at the level of the tissue due to age‐associated changes in the ECM (Amargant et al. 2020; Briley et al. 2016; Dipali et al. 2023; Landry et al. 2022; Umehara et al. 2022). However, aging is also associated with increased stiffness at a cellular level (Phillip et al. 2015). Studies using atomic force microscopy have demonstrated that increased force is required to indent human epithelial cells and fibroblasts with age (Berdyyeva et al. 2005; Dulińska‐Molak et al. 2014). An age‐associated increase in cell stiffness is correlated to changes in the cytoskeleton, in particular increased levels of F‐actin, as well as increased cytoskeletal crosslinking and bundling (Esue et al. 2009; Schulze et al. 2012; Starodubtseva 2011). Age‐dependent changes to cell mechanics may contribute to functional decline given mechanical regulation of cell migration and adhesion (Phillip et al. 2015). Additional studies are required to determine the cell identities of the distinct morphological clusters of ovarian somatic cells, probe cellular stiffness and mechanics in these somatic cell populations, and determine how these cells contribute to ovarian aging.

In our study, we demonstrated upregulation of actin polymerization or depolymerization and actin filament organization pathways in ovarian mesenchymal cells with advanced age, in addition to the presence of morphologically large cells with high actin content. This age‐dependent increase in actin content and organization pathways was coupled with downregulation of cell adhesion and cell migration pathways, as well as phenotypically reduced aggregation of ovarian somatic cells from old mice into organoids. These findings are suggestive of an age‐dependent increase in cell stiffness of ovarian somatic cells. Consistent with this, depolymerization of actin using Latrunculin A shifted ovarian somatic cell morphology and resulted in a partial rescue of organoid formation of cells from old mice. We did not perform this experiment with young mice in parallel because ovarian somatic cells isolated from young mice begin to aggregate efficiently by 1 h post cell seeding and the effect of LatA on aggregation kinetics would be difficult to accurately ascertain in this timeframe. Interestingly, LatA treatment increased the percentage of proliferative cells and decreased the percentage of apoptotic cells present in organoids generated from old mice, suggesting that improved aggregation potential may also result in increased organoid functionality. However, future studies are required to determine the impact of improved organoid aggregation across other functional endpoints. Interestingly, dysregulation of pathways associated with the actin cytoskeleton has also been observed in the human ovary with age, suggesting that cellular stiffness may be implicated in reproductive aging across species (Wu et al. 2024). Further studies are needed to determine the identity of cells with increased cellular stiffness and to elucidate whether they are a cause or consequence of the aging ovarian microenvironment.

Overall, we have developed a robust model system of the mammalian ovarian somatic compartment, which maintains the complex cellular heterogeneity, cellular interactions, and functions seen in vivo. This organoid model can be utilized to interrogate ovarian aging and identify new mechanisms underlying this process. Using this model in addition to single cell transcriptomic and morphological analyses, we have uncovered cellular stiffness as a mechanism potentially underlying ovarian aging for future studies.

## 4 Methods

### 4.1 Animals

Female CD‐1 mice aged 5 weeks and female CD‐1 retired breeders were purchased from Envigo (Indianapolis, IN). Upon arrival to Northwestern University, mice were acclimated for at least 1 week and subsequently used for experiments at 6–12 weeks (reproductively young) or 10–14 months (reproductively old). Mice were housed in a controlled barrier facility at Northwestern University's Center for Comparative Medicine (Chicago, IL) under constant temperature, humidity, and light (14 h light/10 h dark). Upon arrival to Northwestern University, animals were provided water and Teklad Global irradiated chow (2916) containing minimal phytoestrogens *ad libitum*. All mouse experiments were performed under protocols approved by the Institutional Animal Care and Use Committee (Northwestern University, Animal Welfare Assurance Number: A3283‐01, IACUC Protocol ID: IS00013082_IM12) and in accordance with the National Institutes of Health Guide for the Care and Use of Laboratory Animals.

Rhesus macaque (*N* = 2; Age: 18 years, menstrual cycle day 16; Age: 8 years, no menstrual cycle data available) ovarian tissue obtained following necropsy was cut into quarters and submerged in SAGE OTC Holding Media (Cooper Surgical, Trumbull, CT) in 5 mL tubes. Tubes were shipped from Beaverton, OR to Chicago, IL with ice packs to maintain the temperature at 4°C. Upon arrival in Chicago, a piece of tissue was fixed in Modified Davidson's (Electron Microscopy Sciences, Hatfield PA) overnight at 4°C and the remaining tissue was processed for somatic cell isolation. The time between surgery and tissue processing for somatic cell isolation was approximately 20 h. The general care, housing, and use of rhesus macaques for experiments was performed under research protocols approved by the Oregon National Primate Research Center (ONPRC) institutional animal care and use committee.

### 4.2 Ovarian Somatic Cell Isolation

Primary murine ovarian somatic cell isolation and plating were performed as previously described (Rowley, Amargant, et al. 2020; Tingen et al. 2011). Ovarian somatic cells were isolated and pooled from at least five mice per experiment to generate organoids, thereby minimizing impacts from individual animal variation. We did not synchronize the estrous cycle stage of the mice at the time of ovary harvest due to the difficulty in doing so given that reproductively old mice have longer, irregular estrous cycles or are acyclic (Balough et al. 2024). Briefly, mouse ovaries were harvested, separated from the bursa, and placed in Dissection Media composed of Lebovitz's Medium (L15, Gibco, Grand Island, NY) supplemented with 1% fetal bovine serum (FBS, Gibco, Grand Island, NY) and 0.5% Penicillin–Streptomycin (Gibco, Grand Island, NY). To enrich for the extrafollicular stromal fraction, ovaries were mechanically punctured with insulin needles to remove early‐stage follicles, as well as cumulus‐oocyte‐complexes and mural granulosa cells from antral follicles. The remaining stroma‐enriched fraction was dissected into smaller pieces using forceps and incubated in αMEM Glutamax (Gibco, Grand Island, NY) supplemented with 1% FBS, 0.5% Penicillin–Streptomycin, 82 units/mL Collagenase IV (ThermoFisher Scientific, Waltham, MA), and 0.2 mg/mL DNase I (ThermoFisher Scientific, Waltham, MA) for 30 min at 37°C in a humidified environment of 5% CO_2_ in air. To assist with enzymatic digestion, the tissue was triturated every 15 min by pipetting. After 30 min, the enzymatic digestion was quenched with an equal volume of αMEM Glutamax supplemented with 10% FBS. The cell suspension was then filtered through a 40 μm strainer to remove oocytes, follicles, and undigested tissue pieces, pelleted (300×*g* for 5 min at room temperature), washed using RPMI 1640 containing 25 mM HEPES and 2 mM L‐glutamine (Gibco, Grand Island, NY) supplemented with 10% FBS and 1% Penicillin–Streptomycin (Plating Media), and plated. Cells were plated overnight in 2D culture in a differential plating step to remove any oocytes smaller than 40 μm because oocytes do not adhere to tissue‐culture treated plastic and are thus removed during subsequent washes. Following plating overnight, cells were washed with Dulbecco's PBS without calcium or magnesium (DPBS, Gibco, Grand Island, NY) and lifted from 2D culture using 0.05% Trypsin–EDTA (Gibco, Grand Island, NY).

For cell morphology analysis, slides were coated with 50 μg/mL collagen I (Corning, Corning, NY) diluted in DPBS for 90 min at 37°C. Cells were strained through a 40 μm cell strainer and seeded onto collagen‐coated slides at a density of 58,000 cells/slide. Following culture on collagen‐coated slides overnight, cells were treated with 1 μM Latrunculin A (LatA, Sigma‐Aldrich, St. Louis, MO), 1 μM Jasplakinolide (JASP, Sigma‐Aldrich, St. Louis, MO), or vehicle (DMSO, Sigma‐Aldrich, St. Louis, MO) and incubated at 37°C for 45 min. Slides were then washed with DPBS and fixed with 4% Paraformaldehyde (Electron Microscopy Sciences, Hatfield, PA) for 12 min at room temperature.

For isolation of primary somatic cells from rhesus macaque ovarian tissue, ovarian tissue quarters were cut into 500 μm thick slices using a Stadie‐Riggs Tissue Slicer to separate the ovarian cortex and medulla. Tissue slices were placed in Dissection Media and cut into 2–3 × 2–3 mm pieces using a scalpel. Tissue pieces were transferred to αMEM Glutamax supplemented with 0.1% (w/v) bovine serum albumin (BSA, Sigma Aldrich, St. Louis, MO), 1X Insulin‐Transferrin‐Selenium (ITS, Sigma Aldrich, St. Louis, MO), 40 μg/mL Liberase DH (Sigma Aldrich, St. Louis, MO), 82 units/mL Collagenase IV, and 0.2 mg/mL DNase I and incubated for 1 h at 37°C in a humidified environment of 5% CO_2_ in air. To assist with enzymatic digestion, the tissue was triturated every 15 min by pipetting. After 1 h, the digestion was quenched with sterile filtered FBS. The cell suspension was then filtered through a 40 μm strainer, pelleted (300×*g* for 5 min at room temperature), washed using DMEM/F12 (Gibco, Grand Island, NY) supplemented with 10% FBS and 1% Penicillin–Streptomycin, and plated. Following plating overnight, cells were washed with PBS and lifted from 2D culture using 0.05% Trypsin–EDTA.

### 4.3 Organoid Generation and Culture

Organoids were generated using the MicroTissues 3D Petri Dish scaffold‐free, 3D cell culture system (Sigma‐Aldrich, St. Louis, MO, Z764043). Micromolds were cast using 1.5% Agarose (Hoefer Inc., Holliston, MA) dissolved in DPBS and stored at 4°C in DPBS with 1% Penicillin–Streptomycin until use. Agarose micromolds were pre‐equilibrated in 500 μL IntestiCult (StemCell Technologies, Vancouver, CA) at 37°C prior to cell seeding. Following trypsinization, cells were counted using an automated cell counter (Countess, Invitrogen, Waltham, MA), resuspended in IntestiCult at a density of 250,000 cells per 75 μL media, and 75 μL cell suspension was seeded into agarose micromolds. An additional 250 μL IntestiCult was added surrounding micromolds at least 1 h after cell seeding. IntestiCult is a proprietary formula and the composition is unknown. For data shown in Figure 1, HepatiCult (StemCell Technologies, Vancouver, CA), MesenCult (StemCell Technologies, Vancouver, CA), or Plating Media was used to pre‐equilibrate agarose micromolds and resuspend cells as indicated. HepatiCult and MesenCult are proprietary formulas and their compositions are unknown. Media was changed the day following cell seeding and every other day throughout culture.

For TGF‐β treatment, the culture media surrounding agarose micromolds was replaced with IntestiCult containing 10 ng/mL TGF‐β (R&D Systems, Minneapolis, MN) on Day 3 of culture and organoids were cultured for an additional 48 h. Following treatment, organoids were harvested from micromolds, pelleted by centrifugation (500×*g* for 10 min at 4°C), and frozen on dry ice for RNA extraction.

For Latrunculin A and Jasplakinolide treatments, following trypsinization, cells were resuspended in Plating Media containing 1 μM Latrunculin A, 1 μM Jasplakinolide, or vehicle and incubated at 37°C for 30–45 min. Treatments were performed in polypropylene tubes to prevent cell adhesion. Following treatment, cells were pelleted, resuspended in IntestiCult, and seeded into agarose micromolds to assess organoid formation.

### 4.4 Tissue Processing, Histology and Immunohistochemistry

For histology and immunohistochemistry, micromolds were sealed with agarose. Sealed agarose micromolds and mouse ovaries were fixed in Modified Davidson's overnight at 4°C. Following fixation, samples were transferred to 70% ethanol and stored at 4°C until processing. Samples were dehydrated and cleared using an automated tissue processor (Leica Biosystems, Buffalo Grove, IL), embedded in paraffin, and sectioned (5 μm thickness).

Hematoxylin & Eosin staining was performed following a standard protocol. Tissue sections were cleared with Citrosolv (Fisher Scientific Pittsburgh, PA) in 3, 5‐min incubations and mounted with Cytoseal XYL.

Picrosirius Red (PSR) staining was performed as previously published (Amargant et al. 2020; Briley et al. 2016). Briefly, tissue sections were deparaffinized in Citrosolv, rehydrated in graded ethanol baths (100%, 70%, and 30%) and washed in RO water. Slides were immersed in PSR staining solution for 40 min, then incubated in acidified water (0.05 M hydrochloric acid) for 90 s. Tissue sections were dehydrated in 100% ethanol, cleared in Citrosolv, and mounted with Cytoseal XYL.

Hyaluronan‐binding protein (HABP) assays were performed as previously published (Amargant et al. 2020; Rowley, Rubenstein, et al. 2020). Briefly, tissue sections were deparaffinized in Citrosolv, rehydrated in graded ethanol baths (100%, 95%, 85%, 70%, and 50%), and washed in RO water. Endogenous avidin and biotin were blocked using an avidin/biotin blocking kit (Vector Laboratories, Burlingame, CA) and non‐specific antigens were blocked with 10% normal goat serum (Vector Laboratories, Burlingame, CA). Slides were then incubated with biotinylated‐HABP (Calbiochem, San Diego, CA) for 1 h at room temperature. Signal amplification was performed using the Vectastain Elite ABC Kit (Vector Laboratories) and subsequently the TSA (Tyramide Signal Amplification) Plus Fluorescein Kit (1:400; Akoya Biosciences, Marlborough, MA). Slides were mounted with Vectashield containing 4′,6‐diamidino‐2‐phenylindole (DAPI, Vector Laboratories, Burlingame, CA). Slides treated with 1 mg/mL hyaluronidase (Sigma‐Aldrich, St. Louis, MO) were utilized as negative controls.

For chromogenic immunohistochemistry (IHC), tissue sections were deparaffinized in Citrosolv, rehydrated in graded ethanol baths (100%, 95%, 85%, 70%, and 50%), and washed in RO water. Antigen retrieval was performed by microwaving slides in 1X Reveal Decloaker (Biocare Medical, Concord, CA) at 50% power for 2 min followed by 10% power for 7 min. Endogenous peroxidase activity was blocked with 3% hydrogen peroxide, endogenous avidin and biotin were blocked using an avidin/biotin blocking kit, and non‐specific antigens were blocked with 10% normal goat serum and 3% BSA in Tris‐buffered saline (TBS). Slides were then incubated with the respective primary antibody (Table S4) diluted in TBS containing 3% BSA in a humidified chamber overnight at 4°C. Slides were washed in TBS containing 0.1% Tween‐20 (Sigma Aldrich, St. Louis, MO) (TBS‐T) and incubated in secondary antibody (Table S4) for 1 h at room temperature. Signal amplification was performed using the Vectastain Elite ABC Kit and detection was performed using 3,3′‐diaminobenzidine (DAB) with the DAB Peroxidase (HRP) Substrate Kit (Vector Laboratories, Burlingame, CA). Tissue sections were counterstained with hematoxylin, dehydrated in graded ethanol baths (80%, 95%, 100%), cleared in Citrosolv, and mounted with Cytoseal XYL. For immunofluorescent IHC, tissue sections were deparaffinized, rehydrated, and antigen retrieval was performed as described above. Non‐specific antigens were blocked with 10% normal goat serum in TBS containing 0.3% Triton X‐100 (Alfa Aesar, Haverhill, MA). Slides were then incubated with the respective primary antibody (Table S4) in a humidified chamber overnight at 4°C. Slides were washed in TBS‐T and incubated in secondary antibody (Table S4) for 1 h and 45 min at room temperature. Slides were subsequently washed in TBS‐T and signal amplification was performed using the Vectastain Elite ABC Kit and TSA (Tyramide Signal Amplification) Plus Fluorescein Kit. Slides were mounted with Vectashield containing DAPI.

### 4.5 Immunocytochemistry

Following fixation of cells seeded on collagen‐coated slides, slides were washed 3 times with DPBS and cells were permeabilized with 0.01% Triton X‐100 in DPBS for 12 min. Slides were blocked with 0.1% BSA in DPBS for 45 min and then incubated for 1 h at room temperature with a primary antibody against vimentin (Table S4) diluted in 0.1% BSA in DPBS. Slides were then incubated for 1 h at room temperature, protected from light, with goat anti‐rabbit Alexa Fluor 488 (Table S4) and either rhodamine phalloidin (1:50, Invitrogen, Waltham, MA) or Alexa Fluor 633 phalloidin (1:50, Invitrogen, Waltham, MA), and CellMask Orange plasma membrane stain (1:10000, H32713, Invitrogen, Waltham, MA) diluted in 0.1% BSA and mounted in Vectashield containing DAPI.

### 4.6 Dissociation for scRNA‐Seq

To process primary ovarian somatic cells (monolayer) for scRNAseq, cells were strained through a 40 μm cell strainer (PluriSelect, El Cajon, CA) following trypsinization, pelleted at 500×*g* for 5 min, and resuspended in 100 μL DPBS containing 1% BSA. Organoids were harvested from agarose micromolds and dissociated in 0.25% trypsin (Gibco, Grand Island, NY) at 37°C in ultra‐low attachment plates for 1 h while pipetting every 10 min. Trypsin was quenched with Plating Media, cells were strained through a 40 μm cell strainer, pelleted at 500×*g* for 5 min, washed with DPBS containing 1% BSA, and resuspended in 100 μL DPBS containing 1% BSA.

### 4.7 scRNA‐Seq Library Construction and Sequencing

Cell number and viability were analyzed using Nexcelom Cellometer Auto2000 using the AOPI fluorescent staining method. Sixteen thousand cells were loaded into the Chromium iX Controller (10X Genomics, Pleasanton, CA, PN‐1000328) on a Chromium Next GEM Chip G (10X Genomics, Pleasanton, CA, PN‐1000120), and processed to generate single cell gel beads in the emulsion (GEM) according to the manufacturer's protocol. The cDNA and library were generated using the Chromium Next GEM Single Cell 3′ Reagent Kits v3.1 (10× Genomics, Pleasanton, CA, PN‐1000286) and Dual Index Kit TT Set A (10× Genomics, Pleasanton, CA, PN‐1000215) according to the manufacturer's manual. Quality control for constructed library was performed by Agilent Bioanalyzer High Sensitivity DNA kit (Agilent Technologies, Santa Clara, CA, 5067‐4626) and Qubit DNA HS assay kit for qualitative and quantitative analysis, respectively. The multiplexed libraries were pooled and sequenced on Illumina Novaseq6000 sequencer with 100 cycle kits using the following read length: 28 bp Read1 for cell barcode and UMI, and 90 bp Read2 for transcript.

### 4.8 scRNA‐Seq Quality Control and Data Analysis

FASTQ files were generated and demultiplexed from base call (.bcl) files using Cell Ranger from 10× Genomics. Cell Ranger was also used for alignment of the FASTQ files to the mouse reference genome (mm 10) and to count the number of reads from each cell that align to each gene. Resulting matrix files were imported in Seurat (v.4.0), and individual samples were pre‐processed, normalized, and scaled (Hao et al. 2021). Cells with > 7% mitochondrial gene expression, between 500 and 6000 features (unique genes), < 600 or more than 50,000 counts (UMIs), < 19% of all counts from ribosomal markers, and > 0.5% of all counts from red blood cell markers were removed. All samples were combined into a single dataset, with additional metadata containing original sample information. Clusters were separated at the 0.3 resolution using the FindClusters function in Seurat, and differential gene expression analysis was performed to identify biomarkers to define cell clusters and compare between samples. Cell identities were assigned manually based on biomarker genes expressed in each cluster, and endothelial and immune clusters were manually separated. A second dataset was created only using cells from reproductively young and old mice following plating in 2D, but prior to organoid generation (monolayer). Clusters were separated at the 0.4 resolution, and cell identities were assigned manually based on biomarker genes expressed in each cluster. All UMAPs, violin, and raincloud plots were created using tools in Seurat. The CellChat package in R was utilized for cell–cell communication analysis. Integration with the published single‐cell atlas of the aging mouse ovary was performed using Harmony (Isola et al. 2024; Korsunsky et al. 2019). All gene ontology analysis was performed using Enrichr with differentially expressed genes with adjusted *p*‐values < 0.05 and absolute log_2_ fold‐change > 0.58 (Chen et al. 2013). Dot plots were created using the ggplot2 package in R to visualize enriched GO pathways. Related GO terms were grouped manually into broader biological process categories based on the literature.

### 4.9 RNA Extraction and Real‐Time Quantitative PCR (RT‐qPCR)

Total RNA was isolated using a RNeasy Mini Kit (Qiagen, Germantown, MD) with on‐column DNA removal using a RNase‐Free DNase Set (Qiagen, Germantown, MD) following the manufacturer's protocols. A SuperScript III First‐Strand Synthesis SuperMix kit (Thermo Scientific, Waltham, MA) was used to generate cDNA following the manufacturer's protocol. Real‐time quantitative PCR (RT‐qPCR) was performed using PowerUp SYBR Green Master Mix (Applied Biosystems, Thermo Scientific, Waltham, MA) on an Applied Biosystems QuantStudio 7 Pro real‐time PCR machine (Applied Biosystems, Thermo Scientific, Waltham, MA). Melt curves were performed to confirm the presence of a single product per primer pair and positive and negative controls confirmed the quality of the run. Primer sequences were accessed from PrimerBank and are listed in Table S5 (Spandidos et al. 2010). Relative fold‐change of target genes between control and treated samples was determined using the 2^−ΔΔCt^ method (Livak and Schmittgen 2001).

### 4.10 Enzyme‐Linked Immunosorbent Assays (ELISAs)

750 μL conditioned media from organoids generated from reproductively young and old mice was collected at Days 1, 3, and 5 of culture and replaced with 750 μL fresh media. The concentrations of estradiol (E2) and progesterone (P4) in the conditioned culture media were measured using the Estradiol ELISA Kit (Cayman Chemical, Ann Arbor, MI) and the Progesterone ELISA Kit (Cayman Chemical, Ann Arbor, MI), respectively. Assays were performed according to the manufacturer's instructions.

### 4.11 Image Segmentation and Quantification for Cell Morphology Analysis

Fluorescent images of cells from all conditions were acquired with a Leica Stellaris 5 Confocal Microscope at 20× magnification using 3 laser lines (405 Diode, 488 Diode, 568 Diode). Images were taken at 1024 × 1024‐pixel resolution at 0.568 μm per pixel. CellProfiler software was used to segment cells and nuclei from raw fluorescent TIFF images (DAPI was used to identify nuclei and phalloidin or CellMask plasma membrane stain was used to identify cellular structure, particularly in the Lat A treated cells with depleted actin structure). Subsequently, in‐house curation pipelines were used to remove areas of high confluency and ensure single, well‐segmented cells for analysis. The curated masks were leveraged to quantify vimentin and actin content per cell. Specifically, the summation of pixel intensities of fluorescently stained vimentin and actin were quantified per cell. To assess F‐actin texture, the *InfoMeas* parameter of the CellProfiler texture measurement module was analyzed. Over 89,000 single cells spanning both ages and treatments (vehicle, LatA, and JASP) served as the experimental basis for the morphological analysis.

### 4.12 Morphological Feature Extraction

228 morphological parameters were quantified per cell from individual nuclear and cellular masks (features extracted from in‐house pipeline). These morphological parameters ranged from basic geometric features such as area and perimeter to more complex features such as roughness and curvature. To identify key parameters driving variance in the cell sample set, 2D primary factor analysis was performed across all cells and all morphological parameters. 2D primary factor was selected as it would fall in line closer to the subsequent nonlinear 2D UMAP reduction conducted in the paper for selected parameters. Parameters expressing a resulting communality value below 0.5 were dropped for subsequent analysis to clean noise in the dataset (note a higher communality value indicates a parameter capturing more of the variance in a population), resulting in 106 morphological features retained for analysis.

### 4.13 UMAP and k‐Means Morphological Analysis

To compress the 106‐vector morphological space to a more interpretable format, a 2D UMAP transformation was created (reducing the larger vector space to two orthogonal vectors). UMAP is a nonlinear dimensionality reduction algorithm that seeks to capture the structure of high‐dimensional data in a lower‐dimensionality space that keeps spatial relations intact. The morphological dataset was first cleaned through a log transformation and standard scaling (scaling each variable between −1 and 1 and removing magnitude effects across variables). A UMAP model was trained on the preprocessed dataset, creating two constituent vectors (UMAP‐1 and UMAP‐2) with values for each individual cell, allowing for easily interpretable data visualization. Each point in the UMAP space represents an individual cell whose morphological parameters have been transformed and projected. In addition to UMAP, k‐means clustering, an unsupervised hierarchical clustering method, was used to discretize distinct morphological groups within the preprocessed dataset. An optimal number of clusters, 8, was calculated by a plateau in the inertia and silhouette values of the k‐means algorithm. The algorithm was applied to each individual cell, resulting in single cells with unique UMAP‐1, UMAP‐2, and k‐means coordinates.

### 4.14 Imaging and Image Analysis

Transmitted light images of organoids were taken with an EVOS FL Auto Cell Imaging System (ThermoFisher Scientific, Waltham, MA) using a 4× or 10× objective. To view entire agarose micromolds, scans comprised of a series of individual images were taken across the micromold and then automatically stitched together using the EVOS software. Images of tissue sections following histological and chromogenic immunohistochemical staining were taken with a Nikon Eclipse E600 epifluorescence microscope (Nikon, Melville, NY) using a 63× objective, a SPOT Insight 2.0 Megapixel color camera (SPOT Imaging, Diagnostic Instruments, Sterling Heights, MI), and the associated SPOT software (SPOT Imaging, Diagnostic Instruments, Sterling Heights, MI). Images of organoid tissue sections following HABP assays and immunofluorescent immunohistochemical staining were taken with a Leica SP5 inverted laser scanning confocal microscope (Leica Microsystems, Buffalo Grove, IL) using near‐UV (405 nm) and argon (488 nm) lasers. Settings were determined by the Glow‐Over Look Up Table (LUT) using the LAS AF software (Leica Microsystems, Buffalo Grove, IL) to ensure that there was no pixel oversaturation. Epifluorescence images of ovarian tissue sections were taken with an EVOS FL Auto Cell Imaging System equipped with DAPI (Excitation 357/44 nm, Emission 447/60 nm) and GFP (Excitation 470/22 nm, Emission 510/42 nm) LED light cubes using a 20× objective. Imaging settings, including light, gain, and exposure times, were kept consistent between samples.

FIJI software (National Institutes of Health, Bethesda, MD) was used to quantify average area per organoid and average number of organoids per microwell from transmitted light scans. Thresholds were adjusted for each image to capture all cells, and the Analyze Particles function was used to count the number and area of thresholded particles. To quantify the average area per microwell, the total thresholded area was divided by the number of visible microwells. Particles larger than 300 μm^2^ were considered organoids and used for analysis. To quantify the average number of organoids per microwell, the total number of organoids was divided by the number of visible microwells. Organoid area following removal from agarose micromolds was measured manually using the freehand tool in FIJI.

Image analysis following histological and immunohistochemical staining was performed using FIJI software. Total organoid area was measured manually using the freehand tool. To measure positive staining, color deconvolution was performed to isolate the DAB staining from images following chromogenic IHC or the channel of interest was separated from images following immunofluorescent IHC and HABP assays. Thresholds were set based on the highest intensity signal for each marker and kept consistent for all organoids. To calculate percent positive area, thresholded area was divided by the total area for each organoid. Four representative regions of interest (ROIs) in the ovarian stroma and corpora lutea were analyzed for each marker. Total area of ROIs was determined by thresholding and thresholds to capture positive staining were set as described above.

To quantify the number of particles per well following vehicle or LatA treatment, ROIs were drawn over each microwell in transmitted light scans. The total number of particles was measured using the Analyze Particles function following thresholding of each ROI. ROIs containing a majority of cells not within the plane of focus were excluded from analysis.

### 4.15 Statistical Analysis

Statistical analysis was performed using GraphPad Prism Software (La Jolla, CA) or R (RStudio, version 4.3.0). *t*‐tests, one‐way ANOVAs, two‐way ANOVAs (mixed‐effects analysis) followed by Tukey's post hoc test for multiple comparisons, or nonparametric Kruskal–Wallis tests followed by Dunn's multiple comparisons tests were performed to evaluate differences between experimental groups as indicated in the figure legends. *p*‐values < 0.05 were considered significant. All experiments were performed with at least two independent cohorts each comprised of at least five mice and all total organoid or replicate numbers are specified in the figure legends.

## Author Contributions

Shweta S. Dipali, Madison Q. Gowett, Pratik Kamat, Aubrey Converse, Emily J. Zaniker, Jude M. Phillip, and Francesca E. Duncan conceived the project and designed experiments. Shweta S. Dipali, Madison Q. Gowett, Pratik Kamat, Aubrey Converse, Emily J. Zaniker, Abigail Fennell, and Teresa Chou performed experiments and analyzed data. Shweta S. Dipali, Madison Q. Gowett, Pratik Kamat, Aubrey Converse, and Emily J. Zaniker created figures and wrote the manuscript. All authors (Shweta S. Dipali, Madison Q. Gowett, Pratik Kamat, Aubrey Converse, Emily J. Zaniker, Abigail Fennell, Teresa Chou, Michele T. Pritchard, and Mary Zelinski, Jude M. Phillip, and Francesca E. Duncan) edited and approved the final draft.

## Funding

This work was supported by the National Institute of Child Health and Human Development (R01HD093726 to Francesca E. Duncan and Michele T. Pritchard, T32HD094699 to Shweta S. Dipali and Emily J. Zaniker), the National Cancer Institute (F31CA257300 to Shweta S. Dipali), startup funds from the Department of Obstetrics and Gynecology (to Francesca E. Duncan), and P51 OD011092 (DPCPSI, ORIP, NIH) to ONPRC.

## Conflicts of Interest

The authors declare no conflicts of interest.

## Supporting information


**Figure S1:** IntestiCult mouse intestinal organoid growth medium best supports organoid aggregation and growth. (A) Representative transmitted light images of murine ovarian somatic organoids at Days 1, 3, and 5 of culture following seeding of 50,000 or 250,000 cells into the micromold. Scale bars = 400 μm. *N* = 3–4 micromolds per seeding cell density. (B, C) Quantification of the average number of aggregates per microwell (B) and average area per organoid (C) over 5 days of culture for organoids generated from initial seeding of 50,000 or 250,000 cells into the micromold. Error bars represent the standard error of the mean (SEM). *p < 0.05 and ***p < 0.001 by Welch's *t*‐tests at each time point. *N* = 3–4 micromolds per seeding cell density. (D) Representative transmitted light images of murine ovarian somatic organoids cultured in HepatiCult, MesenCult, or RPMI‐1640 media at Days 1, 3, and 5 of culture. Representative images of H&E‐stained murine organoid sections following 5 days in culture in each media. Scale bars for transmitted light images = 400 μm and scale bars for H&E images = 20 μm. *N* = 3–4 micromolds per media type. (E and F) Quantification of the average area per organoid (E) and average number of organoids per microwell (F) over 5 days of culture in each media. Representative images of murine ovarian organoids cultured in IntestiCult, used for comparison, are shown in Figure 1B. Error bars represent the standard error of the mean (SEM). *p < 0.05, **p < 0.01, and ****p < 0.0001 by one‐way ANOVAs at each time point. Asterisk colors indicate significant comparisons. *N* = 3–4 micromolds per media type.
**Figure S2:** Relative abundance of some key cell populations changes in ovarian somatic organoids over time in culture. (A–F) Representative images of vimentin (A, brown), alpha‐smooth muscle actin (⍺‐SMA, B, brown), desmin (C, brown), F4/80 (D, brown), FOXL2 (E, brown), and 3β‐HSD (F, green) IHC staining of mouse ovarian tissue sections and ovarian somatic organoid sections at Days 1 and 6 of culture. For chromogenic staining nuclei were detected by hematoxylin (blue‐purple), and for fluorescent staining nuclei were detected by DAPI (blue). Arrows show F4/80‐positive macrophages restricted to the perimeter of organoids (black, D) and 3β‐HSD‐positive steroidogenic cells excluded from the core of organoids (white, F). Scale bars = 20 μm. Insets are thresholded images highlighting positive pixels. Quantification of the positive area for each marker in regions of interest in mouse ovaries (*N* = 3 ovaries) and ovarian somatic organoids at Days 1 (*N* = 36–45) and 6 of culture (*N* = 39–49). Error bars represent the standard error of the mean (SEM). *p < 0.05, **p < 0.01, and ****p < 0.0001 by Kruskal–Wallis tests followed by Dunn's multiple comparisons tests. (G, H) Additional representative images of 3β‐HSD (green) IHC staining of ovarian somatic organoid sections at Day 1 (G) and 6 (H) of culture showing depletion of 3β‐HSD‐positive cells from the organoid core at Day 6 relative to Day 1. Nuclei were detected by DAPI (blue). *N* = 19 organoids per timepoint.
**Figure S3:** Ovarian somatic cells isolated from rhesus macaque ovarian tissue self‐assemble to form organoids within 24 h. (A) Representative images of medullary primary ovarian somatic cells from rhesus macaque at 0, 1, 2, 16, and 24 h post‐seeding into agarose micromolds. Scale bars = 400 μm. *N* = 1 biological replicate. (B) Representative images of IHC labelling of rhesus macaque ovarian tissue sections using antibodies against Ki67 (brown), cleaved caspase‐3 (CC3, brown), Vimentin (brown), CD68 (brown), and 3β‐HSD (brown). Nuclei were detected by hematoxylin staining (blue‐purple). Scale bars = 2000 μm. *N* = 1–2 biological replicates.
**Figure S4:** Heterogenous cell populations are maintained in ovarian somatic organoids generated from young and old mice. (A) Schematic of samples utilized for single cell RNA‐sequencing (scRNAseq). scRNAseq was performed for two replicates per group and each biological replicate included cells pooled from at least five mice. (B) Separated UMAP plots for young (6–12 weeks) and old (10–14 months) monolayer cells and cells dissociated from young and old ovarian somatic organoids at Day 1 and 6 of culture. (C) Violin plots of a marker gene for each cell cluster for the UMAP plot of primary mouse ovarian somatic cells from young and old mice following plating in 2D, prior to organoid generation (monolayer) and cells dissociated from young and old ovarian somatic organoids at Days 1 and 6 of culture. (D) Table of percentage of each cell cluster for each replicate of monolayer, Day 1 organoid, and Day 6 organoid samples for each age. Bottom row of table includes the total number of cells for each replicate of each experimental group.
**Figure S5:** Epithelial, Luteal 2, and Granulosa 1 clusters exhibit the greatest age‐dependent changes in gene expression. (A) Integrated UMAP plot showing cells colored based on dataset (Red = Dipali et al.; Blue = Isola et al.). (B) Integrated UMAP plots separated by dataset (Red = Dipali et al.; Blue = Isola et al.). (C) Quantification of the number of differentially expressed genes (DEGs) with age within each cluster for cells dissociated from ovarian somatic organoids at Day 6 of culture. scRNAseq was performed for two replicates per group and each biological replicate included cells pooled from at least five mice. (D) Gene ontology (GO) analysis of differentially expressed genes in Epithelial, Granulosa 1, and Luteal 2 clusters with age for cells dissociated from ovarian somatic organoids at Day 6 of culture. Pathways upregulated (up) and downregulated (down) with age are labeled and displayed in manually grouped categories.
**Figure S6:** Cells in the Mesenchyme 1 cluster are matrix fibroblasts. (A) Uniform manifold approximation and projection (UMAP) plot for separate analysis of only primary mouse ovarian somatic cells from young (6–12 weeks) and old (10–14 months) mice following plating in 2D, prior to organoid generation (monolayer). scRNAseq was performed for two replicates per group and each biological replicate included cells pooled from at least five mice. Unbiased clustering revealed 13 distinct cell populations. (B) Violin plots of a marker gene for each cell cluster for the UMAP plot of only primary mouse ovarian somatic cells from young and old mice following plating in 2D, prior to organoid generation (monolayer). (C) Gene ontology (GO) analysis of differentially expressed genes within the Mesenchyme 1 cluster compared to other cell clusters. Biological processes, cellular components, and molecular functions are labeled and displayed in manually grouped categories.
**Figure S7:** Enriched morphological clusters are variable between different groups of old mice. (A) Contour overlays on the morphological UMAP space showing enriched morphology for primary mouse ovarian somatic cells from young (purple) and old (red) mice for 8 independent biological replicates.
**Figure S8:** LatA and JASP treatment impact the morphology of ovarian somatic cells from old mice more than young counterparts. (A, B) Centroid plots visualizing the distance in the UMAP space of each control cell to the average morphological position of all Latrunculin A (LatA, A) and Jasplakinolide (JASP, B) treated cells for each age. Over 89,000 cells spanning both ages (young and old) and all treatments (vehicle, LatA, and JASP) collected from 8 independent biological replicates were utilized for morphological analysis. Box and whisker plots showing quantification of the distance change for LatA (A) and JASP (B) treated cells for each age. ***p < 0.001 by *t*‐tests.
**Figure S9:** LatA treatment improves the age‐dependent impairment in organoid aggregation and JASP treatment reduces the aggregation of ovarian somatic cells from young mice in most replicates. (A, B) Representative images of organoids generated from young and old primary ovarian somatic cells following treatment with Latrunculin A (LatA) or vehicle control at 1 and 9 h post seeding into agarose micromolds for *N* = 2 additional micromolds per condition to that shown in Figure 6A. Scale bars = 500 μm. Right panels for each time point are thresholded masks of cells in agarose micromolds. (C) Representative images of Ki67 (brown) and cleaved caspase‐3 (CC3, brown) IHC staining of vehicle‐treated young, vehicle‐treated old, and LatA‐treated old ovarian somatic organoid sections. Nuclei were detected by hematoxylin staining (blue‐purple). Insets are thresholded images highlighting positive pixels. Scale bars = 20 μm. *N* = 80–81 organoids per condition. (D, E) Quantification of the percent Ki67 (D) and CC3 (E) positive area in vehicle‐treated young (*N* = 80), vehicle‐treated old (*N* = 80), and LatA‐treated old (*N* = 80–81) ovarian somatic organoids after 6 days of culture. Error bars represent the standard error of the mean (SEM). *p < 0.05 and ****p < 0.0001 by Kruskal–Wallis tests followed by Dunn's multiple comparisons tests. (F–H) Representative images of organoids generated from young and old primary ovarian somatic cells following treatment with Jasplakinolide (JASP) or vehicle control at 1 h post seeding into agarose micromolds for *N* = 3 additional micromolds per condition to that shown in Figure 6D. Scale bars = 500 μm. Right panels for each time point are thresholded masks of cells in agarose micromolds.

**Table S1:** Age‐dependent DEGs per cluster for each timepoint.

**Table S3:** Age‐dependent DEGs per cluster in analysis of monolayer only.

**Table S2:** Statistically significant changes in cellular composition of organoids generated from reproductively young and old mice over six days in culture..
**Table S4:** Antibodies used for Immunolabeling.
**Table S5:** RT‐qPCR Primer Sequences (5’‐3’).

## Data Availability

Single cell RNA‐sequencing data files can be downloaded from NCBI Gene Expression Omnibus at accession number GSE273891. Differentially expressed gene lists are available as Supporting Information: Tables S1 and S3.
