# Supplementary material for: Self‐Organizing Ovarian Somatic Organoids Preserve Cellular Heterogeneity and Reveal Cellular Contributions to Ovarian Aging
Source: Aging Cell. 2025 Dec 29;25(1):e70333. doi: 10.1111/acel.70333 (PMC12746702; doi:10.1111/acel.70333)

Supplemental Figure S1

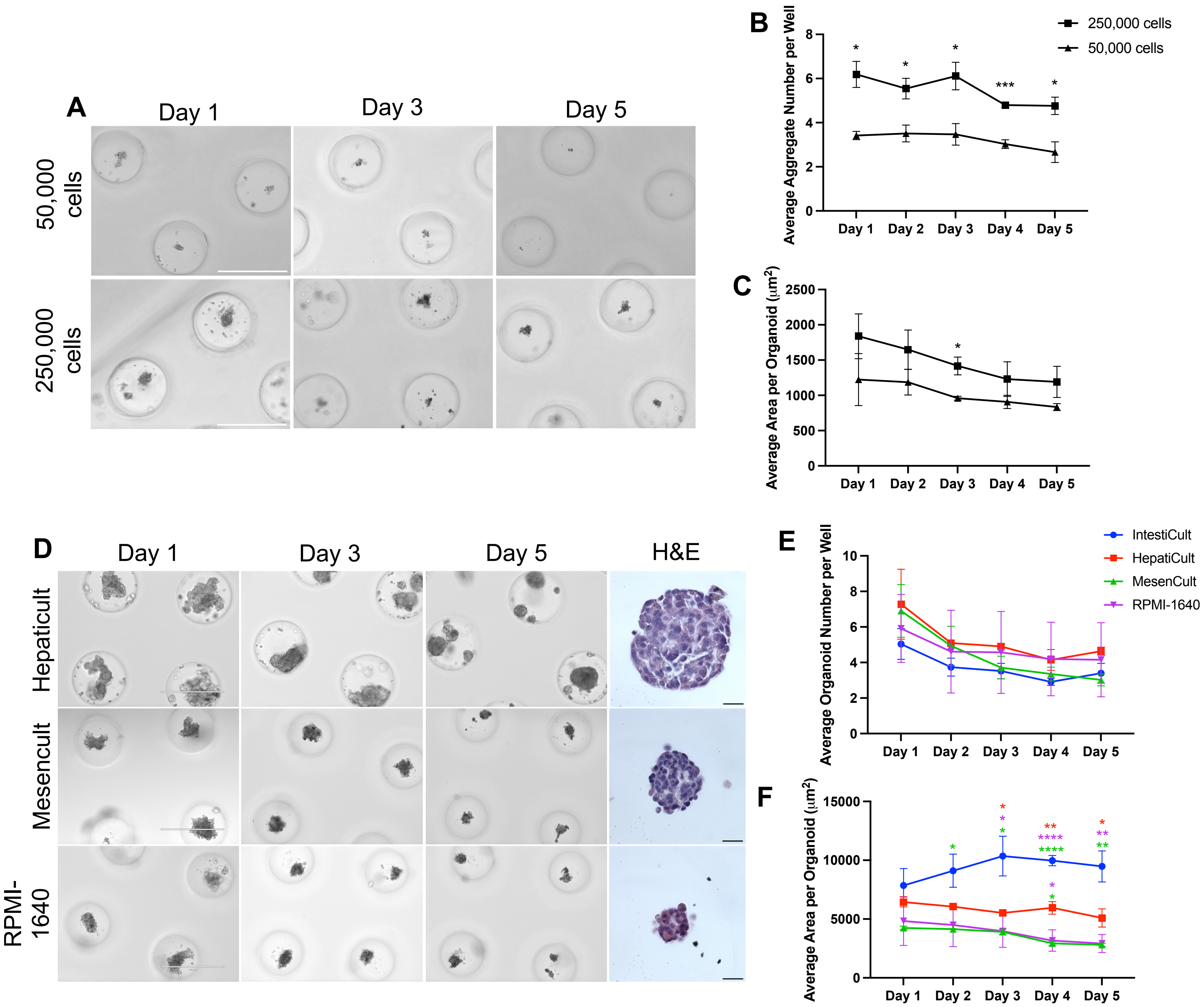

Supplemental Figure S2

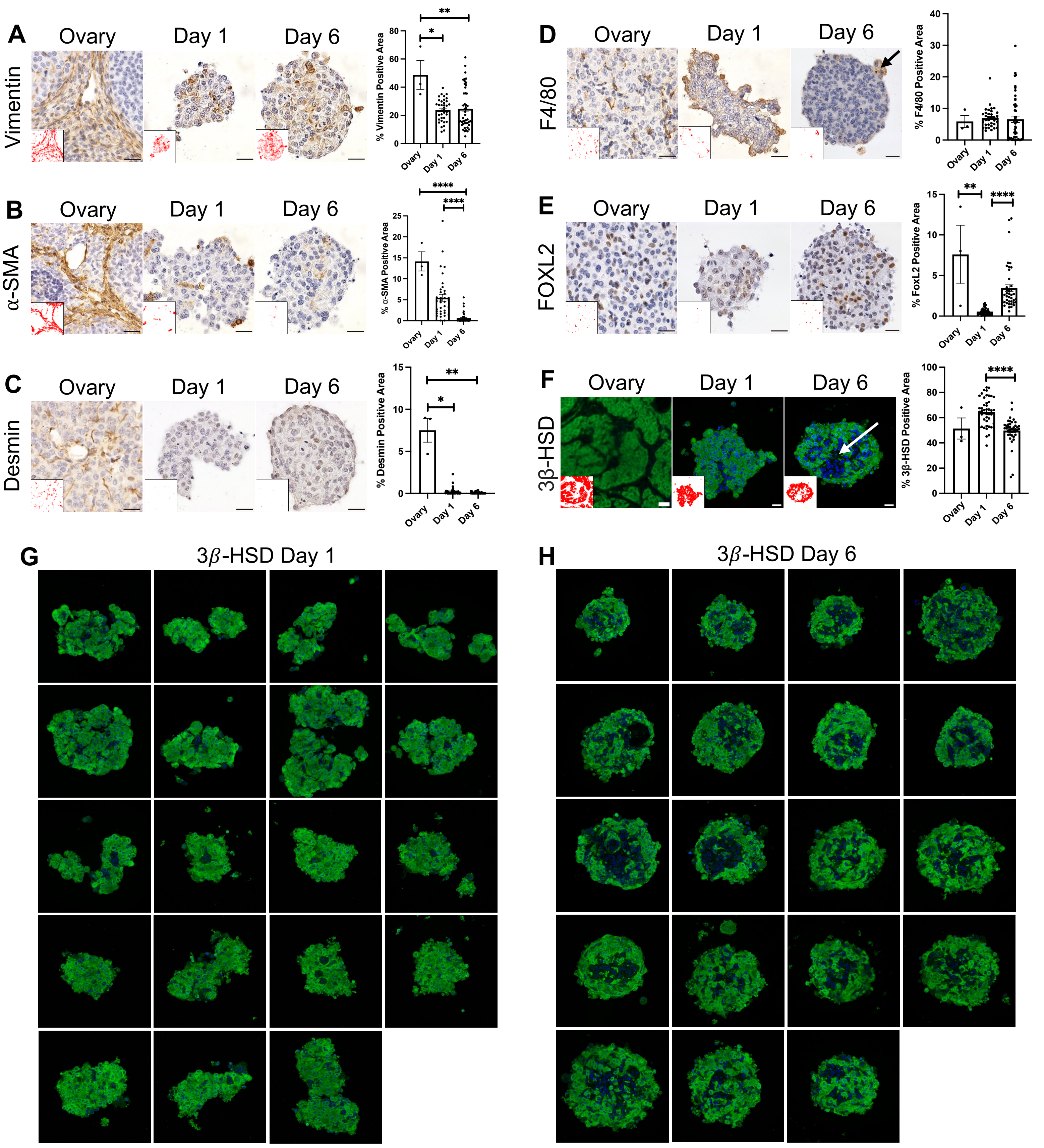

Supplemental Figure S3

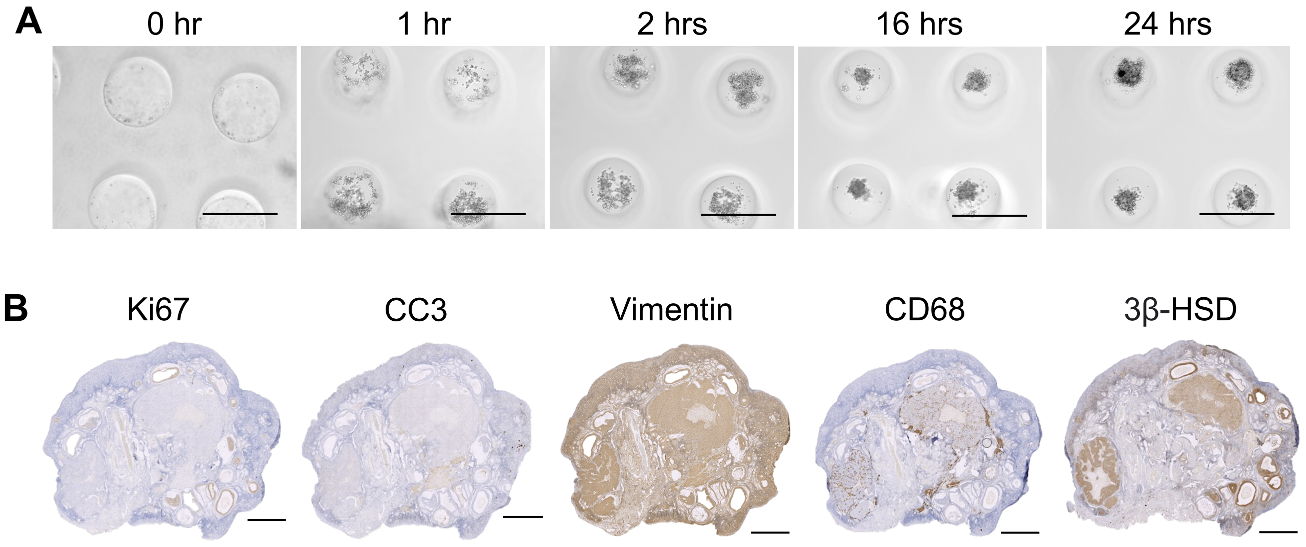

Supplemental Figure S4

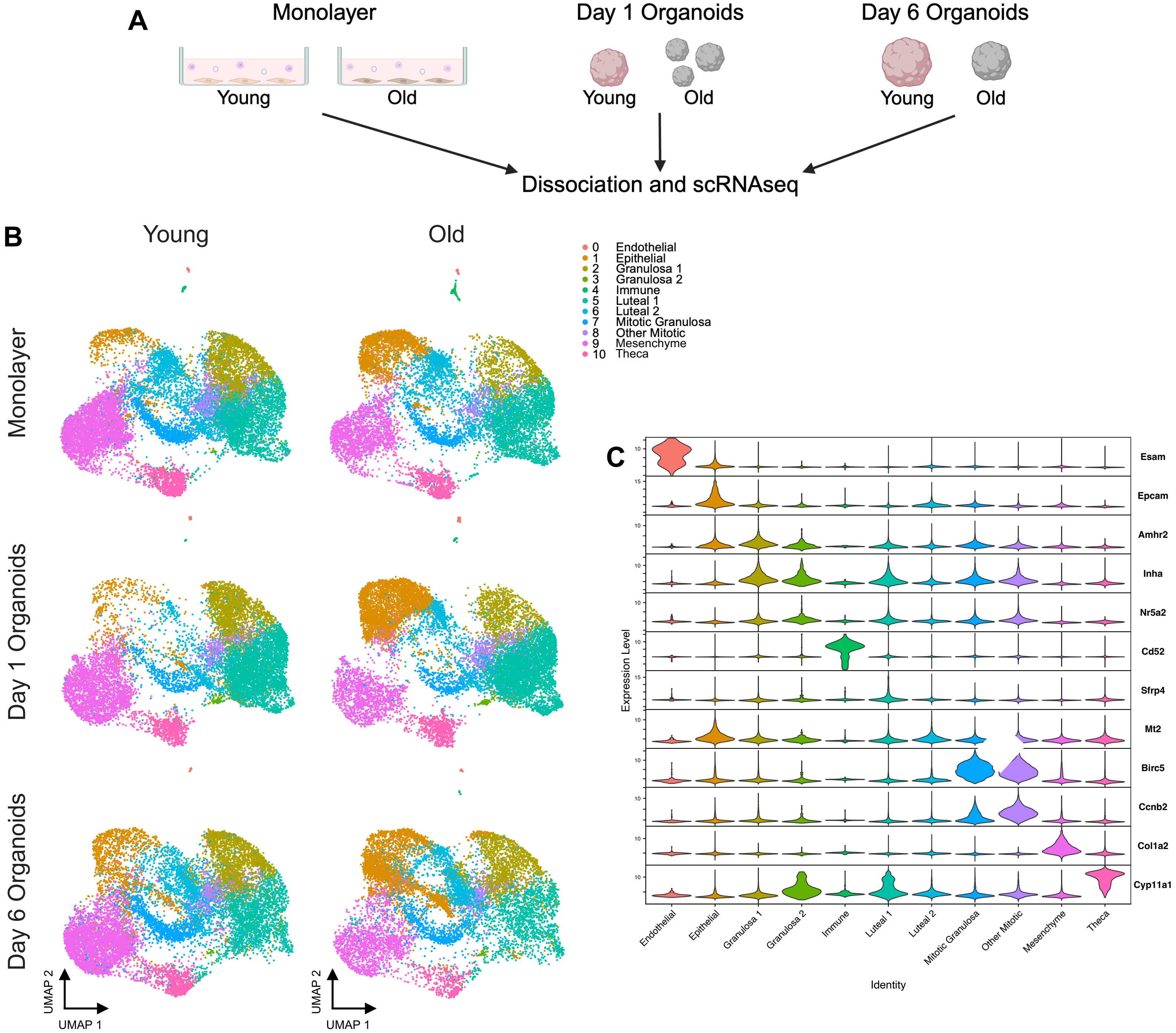

D

Cluster	Young Monolayer		Young D1		Young D6		Old Monolayer		Old D1		Old D6	
	Rep 1	Rep 2	Rep 1	Rep 2	Rep 1	Rep 2	Rep 1	Rep 2	Rep 1	Rep 2	Rep 1	Rep 2
0	0.1%	0.1%	0.0%	0.4%	0.0%	0.1%	0.3%	0.2%	0.3%	0.4%	0.1%	0.1%
1	5.3%	3.1%	4.7%	4.5%	10.4%	7.3%	22.5%	16.5%	19.9%	30.3%	28.0%	29.9%
2	14.3%	14.0%	19.0%	12.7%	17.3%	10.9%	8.7%	8.3%	8.7%	6.2%	13.9%	12.0%
3	0.1%	0.1%	0.7%	1.0%	0.2%	0.1%	0.0%	0.0%	0.6%	0.3%	0.2%	0.6%
4	0.1%	0.3%	0.0%	0.0%	0.0%	0.0%	0.7%	1.5%	0.2%	0.2%	0.0%	0.1%
5	22.3%	19.5%	40.7%	33.8%	11.9%	22.3%	23.5%	26.0%	35.4%	30.3%	12.2%	16.5%
6	14.3%	14.7%	5.5%	7.1%	14.8%	9.8%	16.8%	12.9%	11.7%	7.7%	14.3%	17.7%
7	9.8%	6.3%	4.3%	3.8%	10.7%	5.2%	4.5%	6.9%	3.3%	2.8%	7.7%	5.0%
8	5.6%	4.3%	4.0%	4.0%	5.2%	2.7%	1.6%	3.5%	4.5%	3.3%	3.8%	3.8%
9	22.3%	29.5%	16.7%	26.4%	25.4%	34.1%	14.9%	18.0%	10.1%	13.5%	16.1%	11.3%
10	5.8%	8.1%	4.4%	6.4%	4.1%	7.4%	6.5%	6.3%	5.3%	5.1%	3.7%	3.0%
Total Number of Cells	6698	7175	5062	7235	8987	4490	4042	6952	7123	6347	8103	2861

Supplemental Figure S5

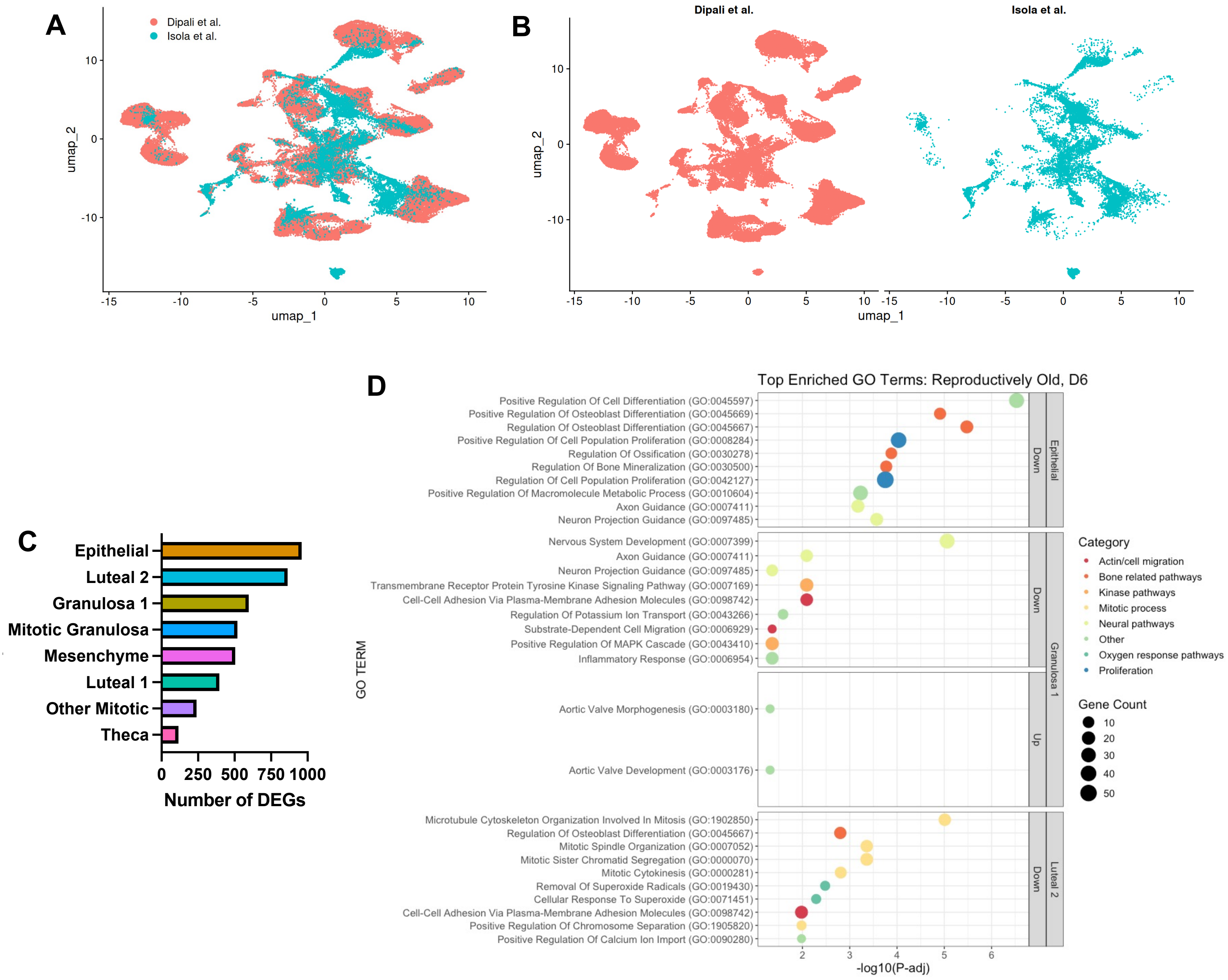

Supplemental Figure S6

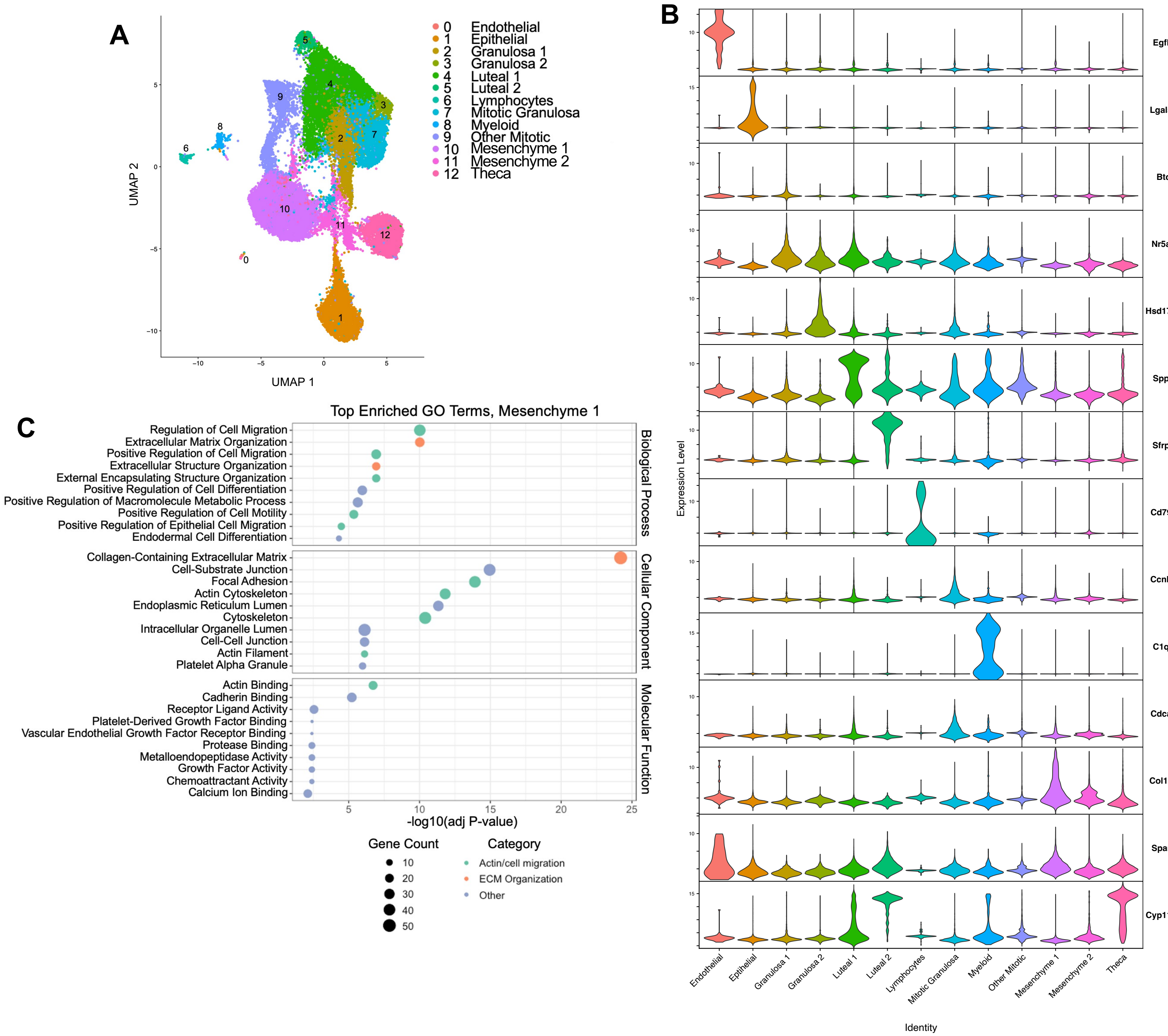

Supplemental Figure S7

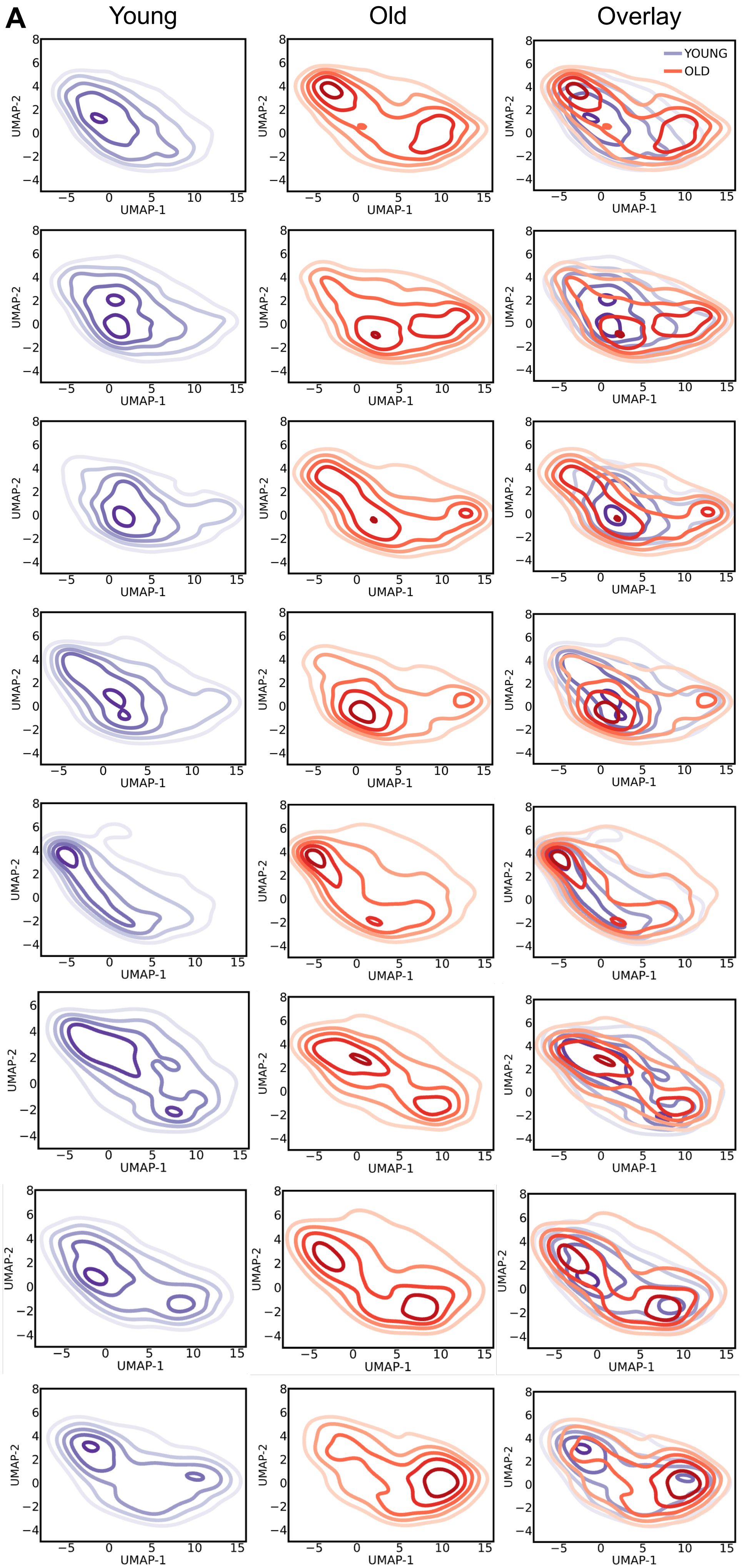

Supplemental Figure S8

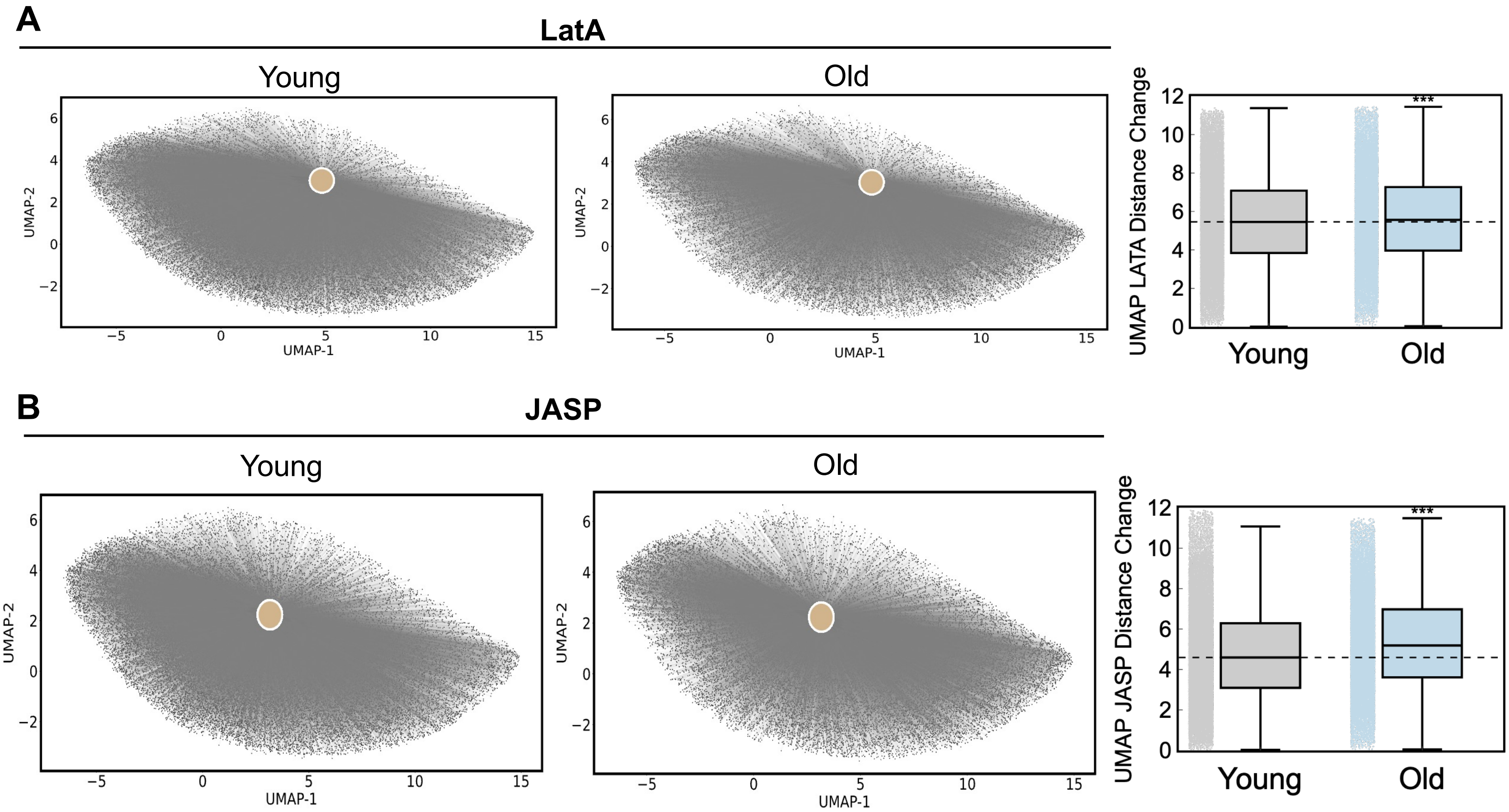

Supplemental Figure S9

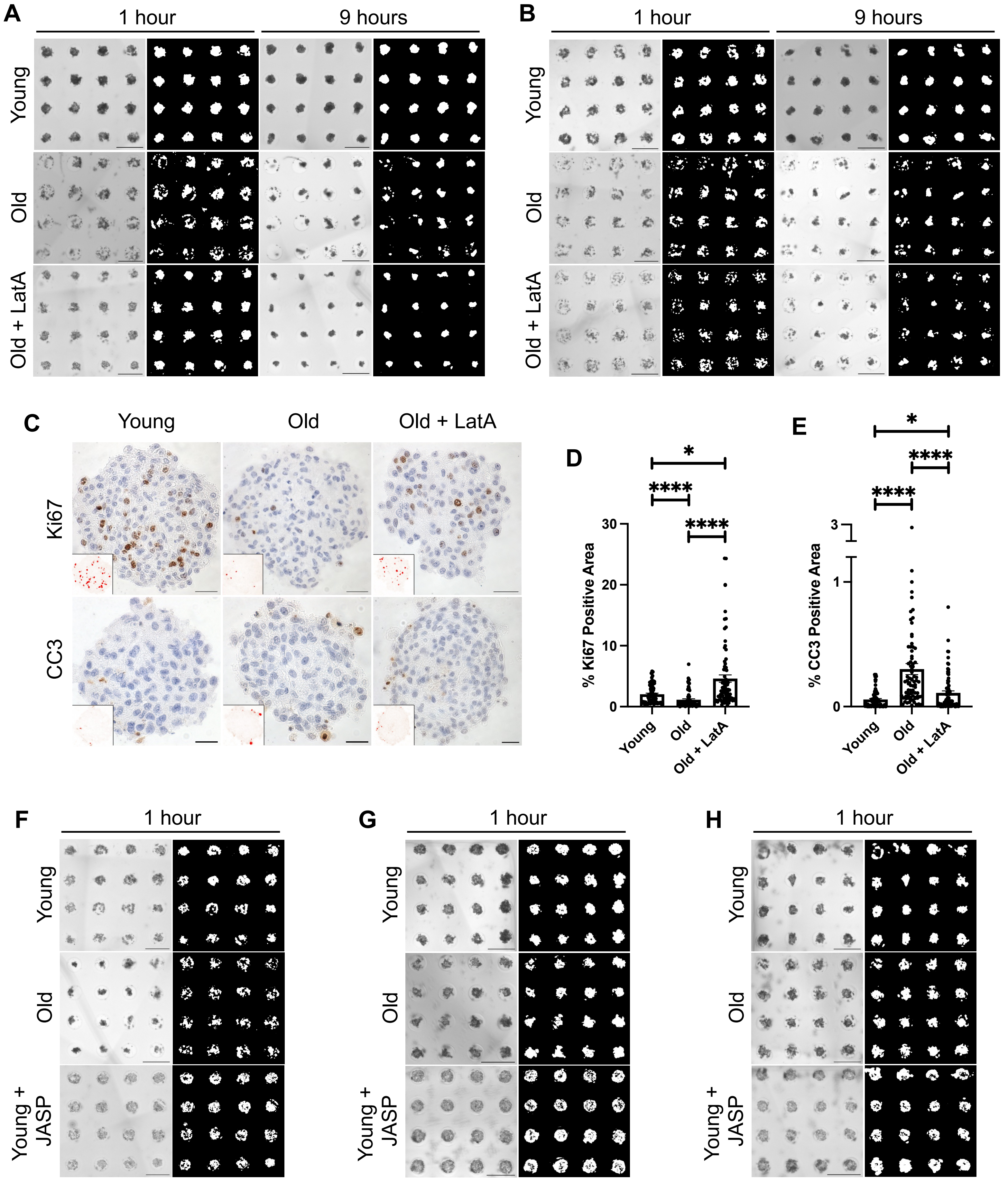

Supplement: Supplementary file 1 — Figure S1: IntestiCult mouse intestinal organoid growth medium best supports organoid aggregation and growth. (A) Representative transmitted light images of murine ovarian somatic organoids at Days 1, 3, and 5 of culture following seeding of 50,000 or 250,000 cells into the micromold. Scale bars = 400 μm. N = 3–4 micromolds per seeding cell density. (B, C) Quantification of the average number of aggregates per microwell (B) and average area per organoid (C) over 5 days of culture for organoids generated from initial seeding of 50,000 or 250,000 cells into the micromold. Error bars represent the standard error of the mean (SEM). *p < 0.05 and ***p < 0.001 by Welch's t‐tests at each time point. N = 3–4 micromolds per seeding cell density. (D) Representative transmitted light images of murine ovarian somatic organoids cultured in HepatiCult, MesenCult, or RPMI‐1640 media at Days 1, 3, and 5 of culture. Representative images of H&E‐stained murine organoid sections following 5 days in culture in each media. Scale bars for transmitted light images = 400 μm and scale bars for H&E images = 20 μm. N = 3–4 micromolds per media type. (E and F) Quantification of the average area per organoid (E) and average number of organoids per microwell (F) over 5 days of culture in each media. Representative images of murine ovarian organoids cultured in IntestiCult, used for comparison, are shown in Figure 1B. Error bars represent the standard error of the mean (SEM). *p < 0.05, **p < 0.01, and ****p < 0.0001 by one‐way ANOVAs at each time point. Asterisk colors indicate significant comparisons. N = 3–4 micromolds per media type. Figure S2: Relative abundance of some key cell populations changes in ovarian somatic organoids over time in culture. (A–F) Representative images of vimentin (A, brown), alpha‐smooth muscle actin (⍺‐SMA, B, brown), desmin (C, brown), F4/80 (D, brown), FOXL2 (E, brown), and 3β‐HSD (F, green) IHC staining of mouse ovarian tissue sections and ovarian somatic organoi [file ACEL-25-e70333-s002.pdf]
